# Joint analysis of the nPOD-Virus Group data: the association of enterovirus with type 1 diabetes is supported by multiple markers of infection in pancreas tissue

**DOI:** 10.1007/s00125-025-06401-x

**Published:** 2025-03-17

**Authors:** Sarah J. Richardson, Teresa Rodriguez-Calvo, Jutta E. Laiho, John S. Kaddis, Julius O. Nyalwidhe, Irina Kusmartseva, Sofia Morfopoulou, Joseph F. Petrosino, Vincent Plagnol, Kathrin Maedler, Margaret A. Morris, Jerry L. Nadler, Mark A. Atkinson, Matthias von Herrath, Richard E. Lloyd, Heikki Hyoty, Noel G. Morgan, Alberto Pugliese, for the nPOD-Virus Group

**Affiliations:** 1 Islet Biology Exeter (IBEx), Department of Clinical and Biomedical Sciences, University of Exeter Medical School, Exeter, UK; 2 Institute of Diabetes Research, Helmholtz Zentrum München, German Research Center for Environmental Health, Munich-Neuherberg, Germany; 3 Faculty of Medicine and Health Technology, Tampere University, Tampere, Finland; 4 Department of Diabetes and Cancer Discovery Science, Arthur Riggs Diabetes and Metabolism Research Institute, Beckman Research Institute, City of Hope, Duarte, CA USA; 5 Department of Medicine, Eastern Virginia Medical School, Norfolk, VA USA; 6 Department of Pathology, Immunology, and Laboratory Medicine, College of Medicine, University of Florida, Gainesville, FL USA; 7 Division of Infection and Immunity, University College London, London, UK; 8 Baylor College of Medicine, Houston, TX USA; 9 Genetics Institute, University College London, London, UK; 10 Centre for Biomolecular Interactions Bremen, University of Bremen, Bremen, Germany; 11 Present Address: Autoimmunity and Primary Immunodeficiency Disease Section, Autoimmunity and Mucosal Immunology Branch, DAIT NIAD NIH DHHS, Rockville, MD USA; 12 UC Davis School of Medicine, Sacramento, CA USA; 13 ACOS-Research Northern California VA Health System, Mather, CA USA; 14 Diabetes Institute, Department of Pathology, University of Florida, Gainesville, FL USA; 15 Diabetes Research Institute, Miller School of Medicine, University of Miami, Miami, FL USA; 16 Fimlab Laboratories, Tampere, Finland; 17 Department of Paediatrics, Tampere University Hospital, Tampere, Finland; 18 Department of Diabetes Immunology, Arthur Riggs Diabetes & Metabolism Research Institute, Beckman Research Institute, City of Hope, Duarte, CA USA

**Keywords:** Autoimmunity, Enterovirus, Pancreas, Pancreatic beta cell, Pancreatic islet, Type 1 diabetes

## Abstract

**Aims/hypothesis:**

Previous pathology studies have associated enterovirus infections with type 1 diabetes by examining the enterovirus capsid protein 1 (VP1) in autopsy pancreases obtained near diabetes diagnosis. The Network for Pancreatic Organ Donors with Diabetes (nPOD) has since obtained pancreases from organ donors with type 1 diabetes (with broad age and disease duration) and donors with disease-associated autoantibodies (AAbs), the latter representing preclinical disease. Two accompanying manuscripts from the nPOD-Virus Group report primary data from a coordinated analysis of multiple enterovirus indices. We aimed to comprehensively assess the association of multiple enterovirus markers with type 1 diabetes.

**Methods:**

The nPOD-Virus Group examined pancreases from 197 donors, recovered between 2007 and 2019, classified into five groups: donors with type 1 diabetes, with residual insulin-containing islets (T1D-ICI group, *n*=41) or with only insulin-deficient islets (T1D-IDI, *n*=42); donors without diabetes who are AAb-negative (ND, *n*=83); and rare donors without diabetes expressing a single AAb (AAb^+^, *n*=22) or multiple AAbs (AAb^++^, *n*=9). We assessed the overall association of multiple indicators of enterovirus infection, case-by-case and between donor groups, as well as assay agreement and reproducibility, using various statistical methods. We examined data from 645 assays performed across 197 nPOD donors.

**Results:**

Detection of enterovirus indices by independent laboratories had high reproducibility, using both enterovirus-targeted and unbiased methods. T1D-ICI donors had significantly higher (*p*<0.001) proportions of positive assay outcomes (58.4%) vs T1D-IDI (10.3%), ND (17.8%) and AAb-positive donors (AAb^+^ 24.6%; AAb^++^ 35.0%). Head-to-head comparisons revealed increased proportions of donors positive in two independent assays among T1D-ICI vs ND donors (VP1/HLA class I [HLA-I], *p*<0.0001; VP1/enterovirus-specific RT-PCR (EV-PCR), *p*=0.076; EV-PCR/HLA-I, *p*=0.016; proteomics/HLA-I, *p*<0.0001; VP1/proteomics, *p*=0.06). Among 110 donors examined for three markers (VP1, EV-PCR and HLA-I), 83.3% of T1D-ICI donors were positive in two or more assays vs 0% of ND (*p*<0.001), 26.7% of AAb^+^ (*p*=0.006), 28.6% of AAb^++^ (*p*=0.023) and 0% of T1D-IDI (*p*<0.001) donors.

**Conclusions/interpretation:**

The nPOD-Virus Group conducted, to date, the largest and most comprehensive analysis of multiple indices of pancreatic enterovirus infections in type 1 diabetes; these were more prevalent in T1D-ICI and AAb^++^ donors than in other groups. Their preferential detection of these indices in donors with residual beta cells and autoimmunity implicates enterovirus infections across disease progression stages and supports a contribution to beta cell loss, directly or indirectly, even after diagnosis. The relatively small number of infected cells and the low amount of viral RNA support the existence of non-acute, low level, possibly persistent enterovirus infections in the pancreas.

**Graphical Abstract:**

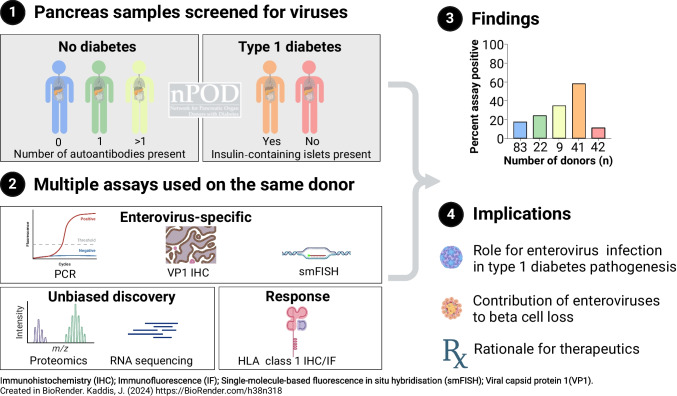

**Supplementary Information:**

The online version contains peer-reviewed but unedited supplementary material available at 10.1007/s00125-025-06401-x.


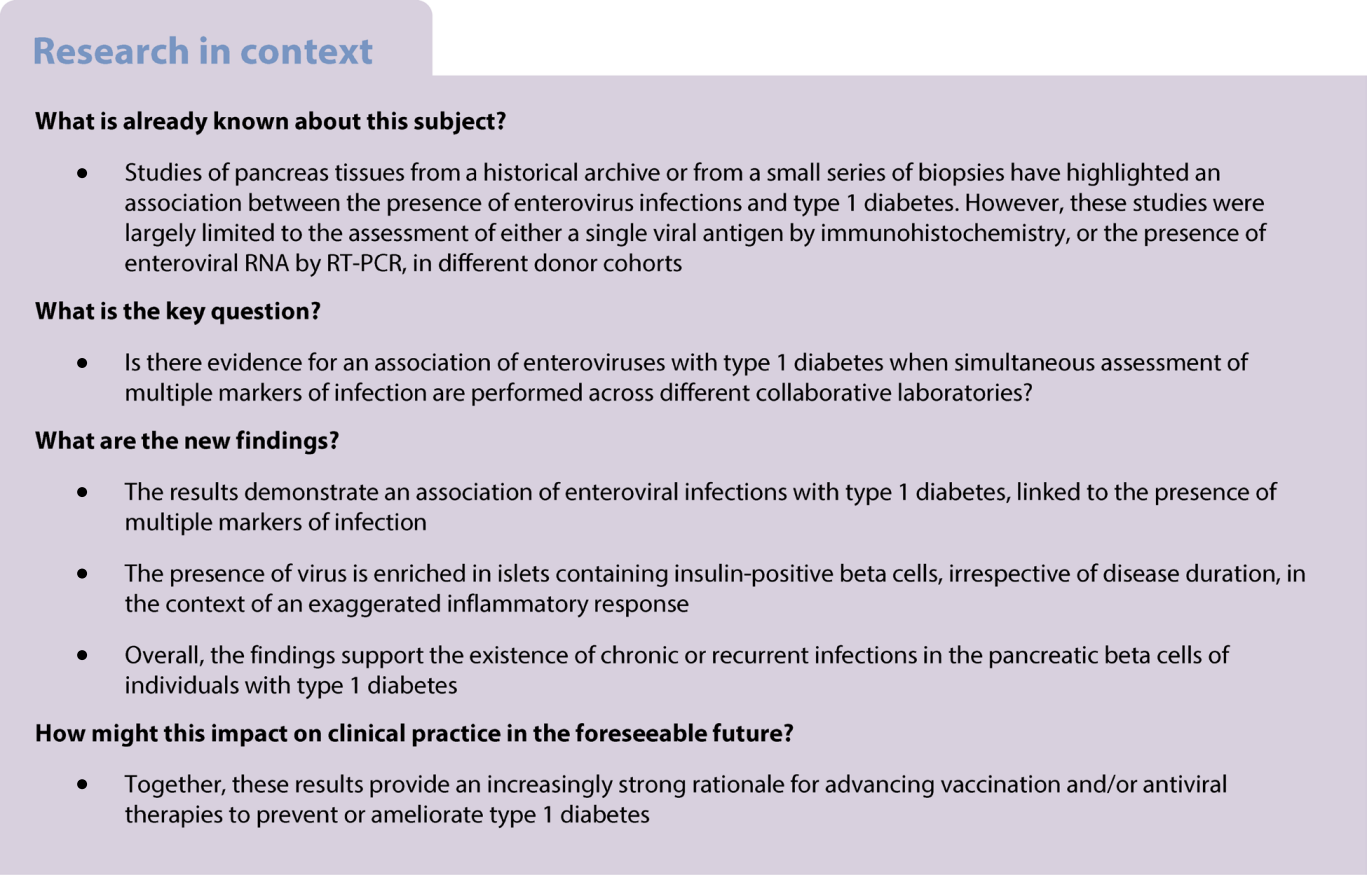


## Introduction

Infectious agents, particularly viruses, have long been suspected to promote type 1 diabetes. Among these, enteroviruses have been implicated by multiple but not all studies; this may reflect assay limitations, suboptimal timing of and frequency of sampling, a highly variable time lapse between infection and diabetes onset, and the high prevalence of enterovirus infections in the general population. Studies involving birth cohorts at genetic risk for type 1 diabetes have overall supported an association between enteroviruses and type 1 diabetes across many populations [1–6]. Supporting evidence includes the following: maternal enterovirus infection during pregnancy is linked to type 1 diabetes risk in the offspring, as confirmed by a meta-analysis of ten studies collectively reporting on 2992 participants (mothers and offspring) [7]; a systematic review and meta-analysis of observational molecular studies of enterovirus infection and type 1 diabetes, which collectively involved 4448 participants, confirming an association [8]; higher rates of enterovirus infection in stool samples of children developing disease [9]; positivity for enterovirus-neutralising antibodies and RNA in serum [10, 11] precedes islet autoantibodies (AAbs) in children at higher genetic risk for type 1 diabetes; AAb^+^ positive children with enterovirus RNA in blood progressed more rapidly to overt diabetes [2]; enterovirus infection, especially prolonged infection, is an independent risk factor for islet autoimmunity in young children with increased genetic risk [12]; and prolonged shedding of the virus may indicate a tendency for persisting infections.

Whether enteroviruses can be found in the pancreas and are associated with type 1 diabetes are challenging questions because access to the pancreas, especially near diagnosis, is rare. Nevertheless, evidence of enterovirus infection has been found consistently in near-diabetes-onset autopsy cases from the Exeter Archival Diabetes Biobank (EADB), pancreas biopsies near diagnosis from the Diabetes Virus Detection (DiViD) study, and an initial, small cohort of organ donors from the network of Pancreatic Organ Donors with Diabetes (nPOD). Across these studies, the enterovirus viral capsid protein 1 (VP1) is detected in a proportion of insulin-positive, residual beta cells at higher frequency in donors with type 1 diabetes than in control individuals: in VP1^+^ donors, 6.9–28.6% of residual insulin-containing islets (ICIs) display VP1^+^ cells and among these 1.5–5.5% of the endocrine cells are VP1^+^ [13–16], across a wide age range [14, 15]. VP1^+^ cells are especially common in islets with insulitis and/or those with hyperexpression of HLA class I (HLA-I) molecules [17, 18]. However, acute, lytic, widespread enterovirus infections have not been reported in the pancreas (except in rare cases with atypical fulminant diabetes [19]), whether in earlier studies of pancreases from EADB autopsies [14], nPOD organ donors [15] or DiViD biopsies [16], all of which included donors with newly diagnosed disease.

Established in 2007, nPOD provides the scientific community with increased access to pancreas from organ donors with type 1 diabetes across a broad age and disease duration spectrum. The nPOD-Virus Group, an international collaboration, was established in 2012 to co-ordinately examine the pancreas and other disease-relevant tissues for signs of viral infection. The group assessed the presence or absence of viral infections, broadly and with a specific focus on enteroviruses, in the pancreas of donors with diabetes or with evidence of islet autoimmunity, and in control donors with neither. Multiple methodologies were deployed, including several not used previously, in separate laboratories, using an unbiased and coordinated (blinded) approach. The primary data from these investigations are presented in three accompanying manuscripts [20–22]. Here, we integrate those findings with other non-overlapping published reports from this group, to present a joint data analysis and comprehensive assessment of an association between type 1 diabetes and markers of enterovirus infection in the pancreas of nPOD donors. Data are derived from assays developed to detect viral proteins (by immunohistochemistry and proteomics) and viral RNA (by RNA-seq, RT-PCR amplification/sequencing, and in situ hybridisation with enterovirus-specific fluorescently labelled RNA probes [single-molecule-based fluorescent in situ hybridisation; smFISH]) [23]. We included a marker of virally induced IFN-α secretion, the hyperexpression of HLA-I molecules by islet cells, which we and others identified as a defining feature of pancreas pathology in type 1 diabetes [18].

## Methods

Our aim was to assess the overall evidence of an association of viral infection with islet autoimmunity and/or type 1 diabetes in pancreatic and other tissues from nPOD donors. To this end, our analysis integrates the results obtained by investigators participating in the nPOD-Virus Group, who examined the presence or absence of markers of viral infection using a variety of approaches, as described in detail in accompanying manuscripts [20–22].

### Donors

Organ donors were obtained by the nPOD (https://npod.org) over a period of 12 years. The data analysis uses the same donor classification as stated in the individual studies. Specifically, donors were classified into five groups: 41 donors with type 1 diabetes and residual insulin-containing islets (ICIs) (T1D-ICI group); 42 donors with type 1 diabetes and only insulin-deficient islets (IDIs) (T1D-IDI group); 83 donors without diabetes who tested negative for islet Aabs (non-diabetic [ND] group; control); 22 donors without diabetes expressing a single AAb (AAb^+^ group); and nine donors without diabetes expressing multiple AAbs (AAb^++^ group). Demographic and clinical features (including sex) are reported in Table 1 and ESM Table 1. All samples were de-identified and obtained by nPOD through its partnership organ procurement organisations, as approved by the University of Florida Institutional Review Board (IRB), after consent for organ donation and research was obtained from family members.

**Table 1:** Summarised donor demographics

Characteristic	ND	AAb^+^	AAb^++^	T1D-ICI	T1D-IDI
No. of donors	83	22	9	41	42
Age, years	22.7 (0.3–75.0)	25.6 (0.2–66.0)	23.0 (17.7–69.2)	22.0 (5.0–79.0)	30.9 (4.4–78.0)
Sex, *n* male/*n* female (% male sex)	51/32 (61.4)	14/8 (63.6)	5/4 (55.5)	19/22 (46.3)	21/21 (50)
BMI, kg/m^2^	24.2 (14.9–41.9)	23.8 (14.8–34.3)	26.0 (19.6–51.4)	24.3 (12.9–42.5)	24.3 (18.4–36.1)
Diabetes duration, years	NA	NA	N/A	5.0 (0.0–56.0)	15.0 (1.5–74.0)
C-peptide^a^, nmol/l	1.49 (0.13–7.58)	1.29 (0.02–8.67)	1.79 (0.17–5.79)		
Donors with detectable C-peptide, *n* (%)				21 (51.2)	2 (4.8)

Data are show as median (range) unless stated otherwise

Individual donor data are provided in ESM Table 1

^a^C-peptide obtained irrespective of fasting/fed state as these are organ donors

### Laboratory methods

**Fig. 1:**
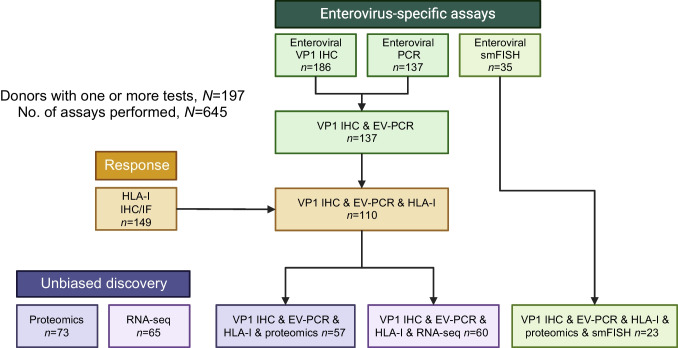
Summary of the types of assays performed and the number of donors assessed per assay or assay combination. Created in BioRender. Richardson, S. (2024) https://BioRender.com/k62i463. IF, immunofluorescence

Multiple assays were used to examine markers of viral infection, as described in the accompanying papers [20–22]. For detailed methods, please refer to ESM Methods. Briefly, at the protein level, assays included immunohistochemistry (IHC) for the assessment of enterovirus VP1 antigen expression, often involving serial pancreas sections (performed in two independent laboratories). IHC and immunofluorescence were deployed to assess the expression of HLA-I molecules, again in two independent laboratories [22] (see ESM Methods: Immunohistochemistry and Immunofluorescence for VP1 and HLA-I). The ‘criteria for enterovirus and HLA-I positivity’ are full described in ESM Methods. Briefly, sections from each donor were evaluated for VP1 staining and classified as VP1-negative (VP1^−^) or VP1-positive (VP1^+^). A donor was considered VP1^+^ in the presence of one or more strongly stained VP1^+^ cell(s) within any islet of a section from either of the participating laboratories. For HLA-I, donors were categorised based on islet HLA-I staining intensities (normal expression, elevated expression or hyperexpression), as previously described [18]. Unbiased, advanced proteomics were used as an independent means to detect and identify viral proteins or peptides [20]. The extraction and processing of protein for MS, LC-MS data acquisition, data processing, database searching and bioinformatic analysis are fully described in the ESM Methods. Pancreas tissues were also used to investigate the presence of viral RNA, using three different methodologies. First, unbiased RNA-seq was undertaken with two different methods developed by two independent laboratories that are fully described in [21] and ESM Methods: ‘Unbiased discovery of microbes’, where ‘RNA-seq analyses’ were performed at University College London (UCL) and ‘metagenomic whole genome shotgun sequencing’ was performed at Baylor College of Medicine (BCM), followed by specific ‘bioinformatic and community profiling’. Second, enterovirus-specific ultra-sensitive RT-PCR was performed as described in [21] and ESM Methods: ‘Targeted enterovirus detection by RT-PCR’, followed by sequencing [24] and [20]. Finally, smFISH using enterovirus-specific RNA probes was carried out on pancreas sections [23]. Several of these methods were jointly developed and refined during the study and some were used for our specific purpose for the first time (e.g. RNA-seq, smFISH and proteomics to detect viral RNA/peptides in pancreas tissue). Figure 1 illustrates the workflow and the number of donors alongside specimen allocation to each of the assay types. These allocations reflected complex logistical considerations, including tissue availability and the specific goals of each analysis. Thus, not all donors could be examined in all assays. Overall, a combined total of 645 assays were performed for 197 nPOD organ donors. Table 2 reports the specific allocation of samples from each donor group to the various assays and details the proportion of donors in each group reporting a positive test in each assay. Across donor groups, we examined 86–100% of donors for VP1 immunopositivity, 4–88% for proteomics, 59–81% for enterovirus-specific RT-PCR (EV-PCR), 64–100% for HLA-I expression, 23–54% for RNA-seq and 2–33% with smFISH. RNA-Seq, smFISH and proteomics assessments were discovery approaches designed to be performed on a smaller scale. The consistent lack of a positive signal in the initial RNA-seq analysis did not support expanding this analysis further. For each assay, detailed results and comparisons across the groups are reported in the companion papers [20–22], which also discuss the limitations and strengths of each assay. The smFISH data have been published but have not been analysed together with the results from the other studies performed by the group [23].

**Table 2:** Allocation of donors to each assay employed and proportion of donors with a positive test for each of the assays

Donor group	VP1 IHC		Proteomics		EV-PCR		HLA-I		RNA-seq		**s**mFISH	
	No. donors examined (%)	No. examined VP1^+^ (%)	No. donors examined (%)	No. examined Proteomic^+^ (%)	No. donors examined (%)	No. examined EV-PCR^+^ (%)	No. donors examined (%)	No. examined HLA-I h/e (%)	No. donors examined (%)	No. examined RNA-seq^+^ (%)	No. donors examined (%)	No. examined smFISH^+^ (%)
Total (*N*=197)	186 (94.4)	79 (42.5)	73 (37.1)	33 (45.2)	137 (69.5)	23 (16.8)	149 (75.6)	46 (30.9)	63 (32.0)	0 (0)	35 (17.8)	10 (28.6)
ND (*N*=83)	76 (91.6)	28 (36.8)	31 (37.3)	12 (38.7)	49 (59.0)	4 (8.2)	54 (65.1)	0 (0)	21 (25.3)	0 (0)	14 (16.9)	0 (0)
AAb^+^ (*N*=22)	19 (86.3)	5 (26.3)	9 (40.9)	4 (44.4)	15 (68.2)	8 (53.3)**	20 (90.9)	2 (10)	12 (54.5)	0 (0)	6 (27.3)	1 (16.7)
AAb^++^ (*N*=9)	9 (100)	4 (44.4)	8 (88.8)	3 (37.5)	7 (77.8)	0 (0)	9 (100)	5 (55.6)***	4 (44.4)	0 (0)	3 (33.3)	2 (66.7)
T1D-ICI (*N*=41)	40 (97.6)	31 (77.5)***	23 (56.1)	14 (60.9)	32 (78.0)	5 (15.6)	39 (95.1)	37 (94.9)***	16 (39.0)	0 (0)	11 (26.8)	7 (63.6)***
T1D-IDI (*N*=42)	42 (100)	11 (26.2)	2 (4.8)	0 (0)	34 (81.0)	0 (0)	27 (64.3)	2 (7.4)	10 (23.8)	0 (0)	1 (2.4)	0 (0)
AI and T1D-ICI (*N*=72)	68 (94.4)	40 (58.8)*	40 (55.5)	21 (52.5)	54 (75)	13 (24.1)*	68 (94.4)	46 (67.6)***	32 (44.4)	0 (0)	20 (27.8)	10 (50.0)**

^*^p<0.05, **p<0.01, ***p<0.001 vs ND (two-sided Fisher’s exact test; significant after false discovery rate [FDR] corrections for multiple comparisons)

### Binary outcome variables

Each donor sample was examined for the presence or absence of virus indicators. The following criteria was used to score each sample: (1) VP1 was deemed positive for >1 VP1^+^ cell (granular cytoplasmic fluorescence staining) within an islet, otherwise negative; (2) HLA-I was positive if hyperexpression present as previously defined [18, 22], otherwise negative; (3) proteomics was positive if single or multiple viral peptides found, otherwise negative; (4) EV-PCR was positive if C_t_ value less than 42 (Tampere laboratory) or PCR amplicon peak size of 115 bp by capillary electrophoresis (Houston laboratory) followed by obtaining an enterovirus sequence by Sanger sequencing, otherwise negative; (5) RNA-seq was positive if viral transcripts were detected, otherwise negative; and (6) smFISH was positive if >15 viral particles were detected (data taken from a previously published report [23]), otherwise negative. If a sample from an individual with type 1 diabetes had more than one islet with more than five insulin-positive beta cells per islet, the donor was labelled as T1D-ICI, otherwise T1D-IDI.

### Statistical methods

For descriptive statistics, percentages are reported for categorical variables while median plus range (minimum–maximum) was used for continuous variables. Differences in negative vs positive assay results between pairings of different donor groups were analysed using Fisher’s exact test, with a Benjamini and Hochberg false discovery rate correction for multiple comparisons. For differences involving multi-class variables, a Pearson’s χ^2^ test or the Freeman–Halton extension of Fisher’s exact test was used; where values from two or more cells were equal to 0, *p* values were not calculated. To evaluate the agreement of results between different assay methods applied to samples from the same individual, Gwet’s AC1 was used to generate estimates of inter-rater reliability [25]. The strength of the agreement was based on the following scale, modified from that originally proposed for the κ coefficient: 0.0–0.2 poor agreement; >0.2–0.4 slight agreement; >0.4–0.6 moderate agreement; >0.6–0.8 good agreement; and >0.8–1.0 excellent agreement [26]. Gwet’s AC1 coefficient 95% CIs and *p* values were also computed. Finally, the negative and positive percentage agreement, including 95% CIs, was determined for each comparison made between assays and a weighted overall percentage agreement was computed based on the number of samples in each category. All statistical analyses were performed using GraphPad Prism 9.3.1 (GraphPad Software, Boston, MA, USA; www.graphpad.com), SAS software, version 9.4 TS Level 1 M3 (SAS Institute, Cary, NC, USA; www.sas.com) or the R programming language, version 3.6.1 (https://cran-archive.r-project.org/bin/windows/base/old/3.6.1/). All software was run on Windows-based machines. An SAS macro written by J. S. Uebersax was modified to calculate positive and negative agreement as well as 95% CIs (http://www.john-uebersax.com/stat/sp_sas.txt). Found in the R package irrCAC, R functions written by K. L. Gwet was used to calculate Gwet’s AC1 agreement coefficient statistics. All *p* values are two-sided and significant if <0.05.

## Results

### Associations of individual viral markers with islet autoimmunity and/or type 1 diabetes

The collective results from each assay are summarised in Table 2 (full results are in [20–22]). T1D-ICI donors were more frequently enterovirus VP1^+^ by IHC (77.5%) compared with ND donors (36.8%), *p*<0.001. Detection of enterovirus peptides using proteomics occurred in 60.9% of T1D-ICI donors; given the smaller number of donors examined with this approach, this higher prevalence was not statistically different from the ND group (38.7%). Hyperexpression of HLA-I was detected by IHC/immunofluorescence in 94.9% of T1D-ICI and in 55.6% of AAb^++^ donors compared with 0% of ND donors (*p*<0.001). Among AAb^+^ donors, 53.3% tested positive using EV-PCR compared with 8.2% in the ND group (*p*<0.001). Although 15.6% of T1D-ICI donors had a positive EV-PCR test, this frequency was not statistically different from that in ND donors. RNA-Seq did not reveal any viral sequences, including enterovirus sequences, in any donor. RNA probes used in the smFISH analysis yielded positive signals in 7/11 (63.6%) T1D-ICI donors and 2/3 (66.7%) AAb^++^ donors compared with none of 14 ND donors (*p*<0.005 vs T1D-ICI donors; *p*=0.01 vs combined T1D-ICI and AAb^+/++^ groups; not corrected for multiple comparisons as only one comparison was made). Therefore, among the individual assays, VP1 positivity and islet cell hyperexpression of HLA-I molecules were the more strongly associated with type 1 diabetes in donors with residual beta cells.

### Associations of combined viral markers with islet autoimmunity and/or type 1 diabetes

**Fig. 2:**
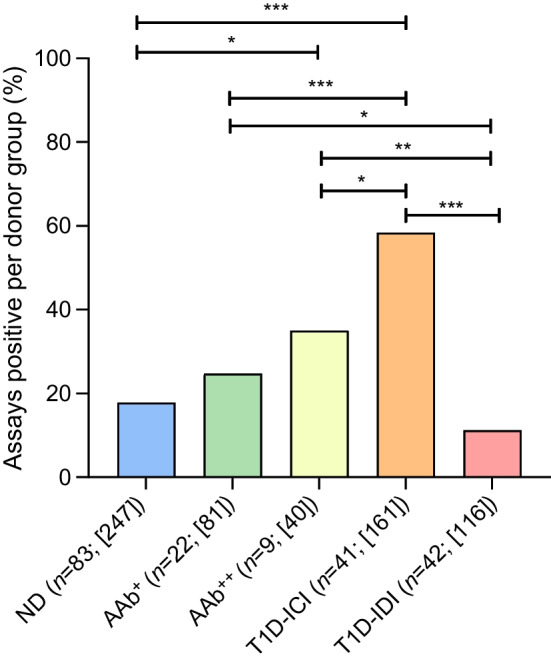
Assessment of total assay positivity across different donor groups. The combination of all 645 assays performed over 197 nPOD donors demonstrates that T1D-ICI donors had a significantly higher proportion of positive assay outcomes when compared with ND, AAb^+^, AAb^++^ and T1D-IDI donors. *p<0.05, **p<0.01, ***p<0.001 (two-sided Fisher’s exact test; significant after false discovery rate [FDR] corrections for multiple comparisons). The total number of assays performed/donor group are shown within square brackets

**Fig. 3:**
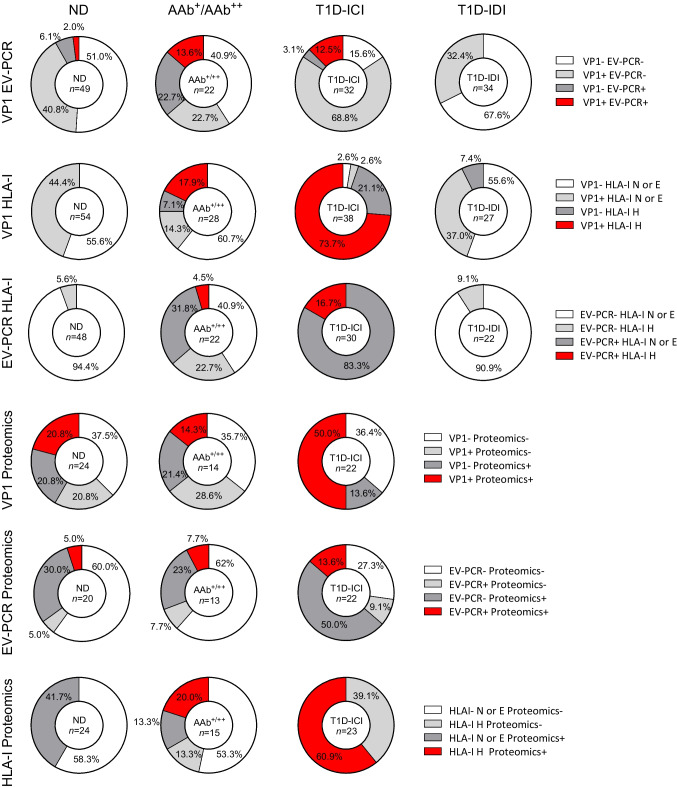
Pairwise comparisons of different assays. Combinations of VP1 and EV-PCR, VP1 and HLA-I, EV-PCR and HLA-I, VP1 and proteomics, EV-PCR and proteomics and HLA-I and proteomics across different donor groups revealed that the T1D-ICI group had an increased percentage of donors who were double-positive (red) for the assays compared with ND donors. The number within each donut represents the total number of donors assessed in that donor group

We then investigated whether any combinations of viral markers were associated with islet autoimmunity and/or disease. Figure 2 illustrates that T1D-ICI donors had a significantly higher proportion of positive assay outcomes (94/161; 58.4%) compared with ND donors (44/247; 17.8%, *p*<0.001), T1D-IDI donors (12/116; 10.3%, *p*<0.001), AAb^+^ (20/81; 24.7%, *p*<0.001) and AAb^++^ donors (14/40; 35.0%. *p*<0.05). Combining the AAb^+^ and AAb^++^ groups revealed increased positivity compared with the ND donor group (*p*=0.03). Figure 3 and ESM Table 2 illustrate which markers were associated with each other at increased frequency in type 1 diabetes when assessed in pairwise combinations. We compared the proportion of donors in each group that were positive for two different markers in the same assays in other donor groups. Higher proportions of double-positivity were observed in the T1D-ICI group vs the ND group for the VP1 and HLA-I (73.7% vs 0%, *p*<0.0001), VP1 and proteomics (50% vs 20.8%, *p*=0.0626), and HLA-I and proteomics (60.9% vs 0%, *p*<0.0001) assays. Overall, the protein-based assays returned higher positivity rates than RNA-based assays, whether examined individually or in pairs (Fig. 3). Considering the pairing of the two enterovirus-specific assays, VP1 and EV-PCR, 12.5% of T1D-ICI donors and 2.0% of ND donors tested positive for both (*p*=0.076). EV-PCR and HLA-I were positive together in 16.7% of T1D-ICI donors vs 0% of ND donors (*p*=0.016). ESM Fig. 1 and ESM Table 3 shows assay combinations that involved the smFISH analysis, performed for a limited number of donors. A higher proportion of donors in the T1D-ICI group vs the ND group were double-positive for VP1 and smFISH (60.0% vs 0%, *p*=0.0016), smFISH and proteomics (40% vs 0%, *p*=0.0351), and smFISH and HLA-I (63.6% vs 0%, *p*=0.0007).

### Positivity for multiple markers of enterovirus infection is associated with islet autoimmunity, type 1 diabetes and the presence of residual beta cells

**Fig. 4:**
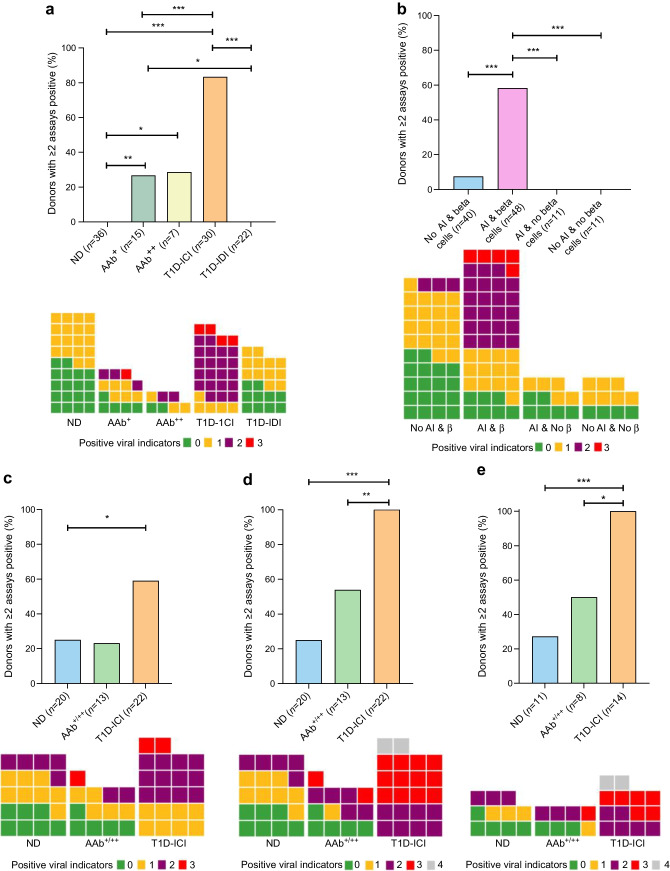
Assessment of positivity in restricted EV-VP1, EV-PCR and HLA-I assays. (**a**) Evidence of autoimmunity and beta cell destruction is associated with an increase in the proportion of donors with positivity in two or more assays. Examination of the 110 donors in which three assays (EV-VP1, EV-PCR and HLA-I) were performed revealed that T1D-ICI donors are significantly more likely to have two or more positive assays when compared with ND, AAb^+^ and T1D-IDI donors. Donors with evidence of autoimmunity also had increased evidence of positivity in two or more assays when compared with ND donors. In T1D-IDI donors, the proportion with two or more assays positive was comparable to that seen in ND donors. *p<0.05, **p<0.01, ***p<0.001 (two-sided Fisher’s exact test; significant after false discovery rate [FDR] corrections for multiple comparisons). The block graphs demonstrate viral indicator counts in ND (*n*=36), AAb^+^ (*n*=15), AAb^++^ (*n*=7), T1D-ICI (*n*=30) and T1D-IDI (*n*=22) groups, with each square representing one individual. (**b**) Comparison of donors with or without evidence of autoimmunity, defined by the presence of circulating AAbs and/or insulitis (AI) with or without beta cells (β). No AAbs/insulitis and beta cells (*n*=40); AAbs/insulitis and beta cells (*n*=48), AAbs/insulitis and no beta cells (*n*=11), and no AAbs/insulitis and no beta cells (*n*=11). ***p<0.001 (two-sided Fisher’s exact test; significant after FDR corrections for multiple comparisons). The block graphs demonstrate viral indicator counts in individuals with no AAbs/insulitis and with beta cells (*n*=40), individuals with AAbs/insulitis and beta cells (*n*=48), individuals with AAbs/insulitis and no beta cells (*n*=11), and individuals with no AAbs/insulitis and no beta cells (*n*=11), with each square representing one individual. (**c**) Proportion of donors with two or more positive assays in whom three enterovirus-specific assays (VP1, EV-PCR and proteomics) were performed; ND (*n*=20), AAb^+/++^ (*n*=13) and T1D-ICIs (*n*=22) donors. The block graphs demonstrate viral indicator counts in ND (*n*=20), AAb^+^ (*n*=6), AAb^++^ (*n*=7), T1D-ICI (*n*=22) and T1D-IDI (*n*=2) donors. (**d**, **e**) Examination of donors in which four assays (VP1, PCR, HLA-I and proteomics; *n*=57) (**d**) and in which five assays (VP1, EV-PCR, proteomics, HLA-I and RNA-seq; *n*=34) (**e**) were performed revealed that T1D-ICI donors were significantly more likely to have two or more assays positive when compared with ND donors or those with evidence of islet autoimmunity (AAb^+/++^). *p<0.05, **p<0.01, ***p<0.001 (two-sided Fisher’s exact test). AI, AAbs and/or insulitis; β, beta cells

Next, we focused on 110 donors who had all been tested for the three most informative assays, VP1, EV-PCR and HLA-I. Collectively, these provide evidence of the presence of enterovirus at the protein and RNA levels and, notably, the HLA-I host response shown specifically associated with VP1 positivity in the accompanying manuscript [22]. In this analysis (Fig. 4a), 83.3% of T1D-ICI donors (25 of 30) were positive for two or more assays compared with none of the ND donors (0 of 36), 26.7% of AAb^+^ (4 of 15) donors, 28.6% of the AAb^++^ (2 of 7) donors and 0% (0 of 22) of the T1D-IDI donors. Multiple statistically significant differences were observed among groups (Fig. 4). T1D-ICI, AAb, and AAb^++^ donors had higher levels of positivity in at least two of the three assays compared with ND donors. The results again showed multiple signs of enterovirus infection more commonly occurring in donors with islet autoimmunity and residual beta cells (Fig. 4b). In contrast, this was rare in T1D-IDI donors.

Combinations of positive markers of viral infection were observed at higher frequencies when subdividing these 110 donors according to their evidence of autoimmunity (circulating AAbs and/or insulitis) and/or the presence of beta cells (Fig. 4b); 58.3% of donors with evidence of autoimmunity and residual beta cells (28 of 48) were positive in >2 assays, vs only 7.5% (3 of 40) of those with residual beta cells but no evidence of autoimmunity, and 0% (0 of 11) of those with ongoing autoimmunity but no beta cells, and 0% (0 of 11) of those with neither autoimmunity nor residual beta cells (Fig. 4b and ESM Table 4). Thus, the presence of multiple markers of viral infection was strongly correlated with islet autoimmunity and the presence of residual beta cells.

Limiting this analysis only to the enterovirus-specific assays, VP1-IHC, EV-PCR and proteomics, where data were available from 57 donors, T1D-ICI donors were positive in two or more assays at a frequency (59.1%) over twice that seen in ND donors (25.0%; *p*=0.03) (Fig. 4c). This dataset was derived from a reduced number of 22 type 1 diabetes cases to match the subset with proteomic analysis; however, it highlights that T1D-ICI donors are more often positive than ND donors for multiple enterovirus-specific markers, regardless of HLA-I hyperexpression. It is especially important that the discovery proteomics approach detected a range of independent enterovirus peptides among these donors, further corroborating the VP1 capsid protein immunopositivity evidence. Across all groups, the detection of enterovirus VP1 by IHC and enterovirus peptides by proteomics were concordant in 33 of the 62 donors examined.

In similar analyses of donors whose tissue was tested in four or five of the assays (Fig. 4d, e and ESM Fig. 2a), T1D-ICI donors exhibited the highest frequency of positivity for multiple markers (two or more assays positive [100%] when compared with ND controls [which ranged from 25 to 27.3%, *p*<0.0003 to *p*=0.0006, respectively]). These differences were observed despite the smaller number of donors included in these comparisons. The addition of smFISH to an extended enterovirus-specific assay panel, which included smFISH, proteomics, EV-PCR and VP1 in ND (*n*=11) and T1D ICI (*n*=9) donors, revealed that all T1D-ICI donors were positive for two or more assays, with 11.1% scoring positive for all four (ESM Fig. 2b).

### Agreement analysis

We assessed the extent of concordance between all pairs of assays for each sample analysed (ESM Table 5). Statistically significant agreement between pairs of assays ranged from poor (Gwet’s AC1<0.00) to almost perfect (AC1>0.80–1.00). Many factors may have affected the level of agreement, including limited matching of pancreas blocks, especially when paraffin and frozen blocks were used in different assays, heterogeneity of sample types, low expression levels (often close to the sensitivity of the assays) and inherent differences in the pathology of individual disease groups. When examining the agreement by donor groups, certain assays were almost perfectly correlated. For example, in ND donors, there was essentially an almost perfect agreement between HLA-I and EV-PCR assays (AC1=0.94, 95% CI 0.85–1.00, *p*<0.0001, *n*=36 samples), resulting in 97% negative (95% CI 93–100%) and 94% overall agreement. This assay pair is highlighted since both assays were performed on a large number of donors and displayed robust sensitivity. In AAb^+/++^ donors, agreement was substantial between the HLA-I and VP1 assay data (AC1=0.64, 95%CI 0.33–0.95, *p*=0.0001, *n*=28), resulting in 85% dual-negative (95% CI 73–97%) and 63% dual-positive (95% CI 35–90%), yielding 79% overall agreement. A similar outcome was also observed in T1D-ICI donors (AC1=0.69, 95% CI 0.46–0.92, *p*<0.0001, *n*=38), resulting in 18% dual-negative (95%CI 12–49%), 86% dual-positive (95% CI 77–95%), and 76% overall agreement. In T1D-IDI donors, strong agreement was seen between the HLA-I and EV-PCR assay data (AC1=0.90, 95% CI 0.75–1.00, *p*<0.0001, *n*=22) where all the samples tested were negative in each assay.

**Fig. 5:**
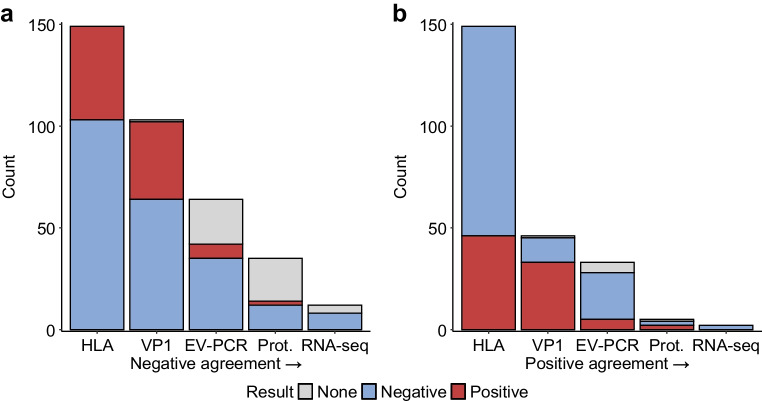
Sequential assessment of assay agreement. HLA-I assay results were evaluated sequentially by four other methods. The number of donors that tested positive (red) or negative (blue) for the assays listed on the *x*-axes are shown. Excluding the donors in the grey boxes, for whom data for certain assays were not available, (**a**) illustrates negative agreement and (**b**) shows positive agreement. As an example, in (**a**), from left to right, the negative agreement for each test being negative is reflected by the extent of the overlap in the blue colour. In this sequential analysis, the agreement is visualised across the different bars. As described in the main text, the highest agreement, whether negative or positive, was found between HLA-I hyperexpression and enterovirus VP1. No samples tested positive by all five assays. All donor groups were included in this analysis. Arrow indicates order of assessment. Prot., proteomics

We then evaluated the HLA-I assay results sequentially, independently of disease status, to establish the extent of agreement among four other methods. Figure 5 plots the number of donors analysed for each assay and aids in visualising the extent of agreement across assays. Regarding negative agreement (Fig. 5a), there was 77% negative agreement of VP1 with HLA-I (95% CI 70–84%; *n*=64) based on 46 and 103 donor samples with/without HLA-I hyperexpression, respectively. Of those HLA-I and VP1 negative, 91% agreement was reached by EV-PCR (95% CI 84–98%; *n*=35). Of those HLA, VP1 and EV-PCR negative, there was 92% agreement by proteomics (95% CI 82–100%; *n*=12). Of those negative for all four assays, there was 100% agreement by RNA-seq (*n*=8). In terms of positive agreement (Fig. 5b), there was 85% positive agreement of VP1 with HLA-I (95% CI 76–93%; *n*=33). Of those HLA-I and VP1 positive, there was 30% agreement by EV-PCR (95% CI 10–51%; *n*=5). Of those HLA, VP1 and EV-PCR positive, there was 67% agreement by proteomics (95% CI 23–100%; *n*=2). No samples tested positive by all five assay methods. Overall, these analyses support an association of HLA-I hyperexpression with enterovirus infection and help collectively define the strengths and limitations of our results.

## Discussion

For decades, a critical question has existed as to whether viruses, particularly enteroviruses, might play a role in the pathogenesis and aetiology of type 1 diabetes. This topic has been addressed extensively in multiple studies, without universally concordant outcomes; yet, the overall evidence for an association between diabetes and enterovirus infections has become ever more persuasive [7, 8, 12, 27, 28]. Perhaps the most compelling association was generated in the TEDDY study, where enterovirus B species, particularly coxsackie B viruses (CVBs), were strongly associated with increased risk of islet autoimmunity and longitudinal assessments demonstrated prolonged shedding of the virus into stools as a marker of delayed clearance of the virus [12]. Further compelling evidence was obtained in a recent clinical trial in which 6 months of treatment with antiviral drugs starting near the diagnosis of type 1 diabetes resulted in preservation of insulin secretion when compared with placebo [29]. These observations suggest that enterovirus infections may play a role in type 1 diabetes and that the virus may persist in children developing disease.

Based on the above associations, it is critical to ascertain whether enteroviruses infect the pancreas, and more specifically beta cells, since such infections may trigger beta cell dysfunction, inflammation and/or islet autoimmunity. Another critical question is whether pancreatic enterovirus infections occur acutely in type 1 diabetes or are chronic (or if both are seen). Such considerations make the timing of detection a critical issue to address. In 1979, coxsackievirus B4 (CVB4) was isolated from the pancreas of a child with recently diagnosed type 1 diabetes. After isolation, this virus infected the beta cells of mice, causing islet inflammation, beta cell necrosis and overt hyperglycaemia [30]. A study in 2007 identified CVB4 in the pancreas of three individuals; after extraction, the virus could infect human beta cells [13]. Questions about these earlier reports have been raised, not least whether the enteroviruses isolated are wild-type or laboratory strains [31]. However, the study of pancreas biopsies from six adults with recent-onset type 1 diabetes [32] in the Norwegian DiViD study provided support for the presence of enterovirus infection near diagnosis, with enterovirus capsid protein VP1 being detected in beta cells (in parallel with marked islet HLA-I hyperexpression) in all six individuals [16] and enterovirus RNA being demonstrated by RT-PCR and nucleic acid sequencing in the islets of four [16]. Moreover, upon co-culture of pancreatic homogenates with enterovirus permissive cell lines, slowly replicating enteroviruses were detected in all six individuals [33]. RT-PCR often detected enterovirus RNA in other tissue samples from these individuals, including duodenum, stools and peripheral blood mononuclear cells, although not in serum, as would be expected in the case of an acute infection [34]. Overall, the data from the DiViD study provide firm support for the hypothesis that a chronic, persistent infection of the pancreas can occur in recent-onset type 1 diabetes, possibly sustained by a slowly replicating strain of enterovirus.

Studies of the pancreas from a larger number of individuals with type 1 diabetes were conducted using the EADB, comprised of formalin-fixed, paraffin-embedded sections from organs obtained post-mortem, at autopsy, soon after the time of diabetes diagnosis. This biobank includes pancreas specimens collected as long ago as the 1940s and has yielded evidence of VP1 immunopositivity in a small proportion of residual beta cells among individuals with type 1 diabetes, while tenfold fewer equivalent control pancreases were immunopositive [13–16].

The present work, together with the accompanying studies, describes the collaborative analysis of pancreas specimens and other disease-relevant tissues obtained from organ donors collected by nPOD in the USA. The nPOD-Virus Group includes investigators from the European PEVNET Consortium, representing a truly global collaboration. The study represents the largest and most comprehensive analysis of pancreas tissue ever undertaken to examine the association between enterovirus infection and type 1 diabetes, through the unique deployment of coordinated, diversified approaches by multiple laboratories examining pancreas tissue from organ donors with and without disease across a spectrum of donor ages, disease stages and disease duration. Collectively, the nPOD-Virus Group has examined pancreatic tissue from 197 nPOD organ donors, collected during a 12 year period, representing a large, contemporary, cohort. Unique to the nPOD cohort, this includes samples from donors with no clinical disease but whose AAb status is consistent with preclinical stages of type 1 diabetes, during which aetiological factors are more likely to be detected.

Our integrated analysis evaluates the association of enterovirus with type 1 diabetes by incorporating multiple markers of infection: the T1D-ICI organ donor group has the highest proportion of positive enterovirus assay outcomes (60%), relative to the other groups studied (range 10–38%, *p*<0.05–0.001). Donors with islet autoimmunity (defined by the presence of circulating islet AAbs and/or insulitis) also had an elevated proportion of positive markers indicating the presence of enterovirus. Overall, 47.5% of donors with autoimmunity and residual beta cells expressed multiple markers of viral infection, whereas no control donor had a similar array of positivity (*p*<0.0001). Our results suggest that enterovirus infections are associated with islet autoimmunity, either at the preclinical or post-diagnosis stages, and importantly correlate strongly with the presence of residual beta cells. This supports the concept that beta cells are an important target of enterovirus infections. While the frequency of VP1^+^ beta cells is low, there is an increased proportion of VP1-immunopositive cells in islets compared with the surrounding exocrine pancreas, as shown in the accompanying paper by Rodriguez-Calvo et al [22]. In an earlier report we showed that an isoform of the coxsackie-adenovirus receptor is selectively expressed by pancreatic beta cells on the intraluminal side of the secretory granules, possibly favouring the infection of beta cells during exocytosis when the receptor becomes exposed to enteroviruses located within the surrounding interstitial space [35].

We evaluated the concordance of differing pairs of assays. Positivity for both VP1 and EV-PCR, representing the two most enterovirus-specific assays used in the study, was most frequent in T1D-ICI (12.5%) donors and was rarely seen among ND donors (2%, *p*=0.07). Positivity for EV-PCR and HLA-I hyperexpression in the same individual was more common among T1D-ICI donors (16.7%) than ND donors (0%, *p*=0.016). The highest double-positivity rates were also observed in the T1D-ICI group for the VP1 and HLA-I combination (73.7% vs 0%, *p*<0.0001 vs ND); HLA-I hyperexpression was similarly associated with the detection of enterovirus peptides by proteomics (60.9% double-positive in T1D-ICI donors compared with 0% among ND donors, *p*<0.0001). Importantly, our proteomic assays detected multiple enterovirus peptides arising from diverse viral proteins; these included the capsid protein VP1 and specifically the epitope recognised by the 5D8/1 detection antibody, supporting the fidelity of our VP1 detection assay. The finding that proteomics detected peptides from enterovirus non-structural proteins suggests that the virus is actively replicating in the pancreas, as non-structural enterovirus proteins are produced only in infected cells but are not incorporated into the final mature virion [20].

Based on the above, we further analysed data from 110 donors who had all been examined for the three markers that in our collective studies had the strongest association with enterovirus infection: VP1 by IHC, enterovirus RNA by RT-PCR and HLA-I hyperexpression; the latter represents indirect evidence of infection and was clearly associated with VP1 positivity in our study [22]. Together, these data provide strong evidence of enterovirus infection at the protein and RNA levels, and of a concomitant host response that is known to be induced by IFN signals evoked by viruses. Once again, T1D-ICI donors exhibited the highest frequency of positivity in at least two of these three assays (83.3%) by comparison with any of the other groups. ND donors had the lowest frequency (0%, *p*<0.0001). AAb^+^ (26.7%) and AAb^++^ (28.6%) donors showed increased proportions of donors with multiple positivity compared with the ND group (*p*<0.05). One of the 15 (6.7%) AAb^+^ and five of the 30 (16.7%) T1D-ICI donors were positive for all three assays, in contrast to none of the ND, AAb^++^ or T1D-IDIs donor groups (Fig. 4a, b).

Similar results were obtained when examining the two enterovirus-specific assays in combination with the specific detection of enterovirus proteins by unbiased proteomics, regardless of the extent of HLA-I expression, since triple positivity was more frequent in T1D-ICI donors. Significant associations were also observed when data were examined from either four of the assays deployed or all five, even if these were inevitably limited to fewer donors. Thus, much like the well-known association of increased risk of type 1 diabetes with positivity for multiple AAbs (which occurs only rarely in healthy individuals), we found that multiple markers of enterovirus infection are specifically associated with type 1 diabetes; this association was not seen in AAb^−^ donors without diabetes. Moreover, markers of viral infection are most common in donors with type 1 diabetes with residual beta cells or in donors without diabetes who were AAb-positive, which further links enteroviral infection with chronic islet autoimmunity.

Unlike earlier studies which examined pancreas from individuals with recent-onset diabetes among the UK EADB autopsy samples [13–16] and the Norwegian DiViD biopsies [16, 32], the nPOD cohort includes individuals with a broad range of ages at diagnosis and disease duration from the USA. Furthermore, the nPOD cohort includes donors at increased risk for type 1 diabetes, which allows for investigation of the prevalence of enterovirus markers at preclinical stages of disease progression. In common with results from EADB and DiViD, we did not identify acute infections even among the nine donors examined within 1 year of diagnosis (*n*=4, <1 month; *n*=2, 1–6 months; *n*=3, 6–12 months). This does not exclude that an initial acute infection had occurred but demonstrating this would require tissue sampling that is coincidental with the short-lived, acute phase of the infection and may require quite extensive tissue analysis as only a small number of islets may be affected. Alternatively, an initial acute infection may occur in a different organ. Regardless of an acute phase of infection, the detection of enterovirus markers for years after diagnosis in the presence of residual beta cells suggests that a low level, possibly persistent enterovirus infection of beta cells is associated with the development of islet autoimmunity and type 1 diabetes. However, since our studies also detected the presence of non-enterovirus viral peptides together with or independent of those from enteroviruses [20], we cannot exclude the possibility that enteroviruses may act together with other infections or that other infections may also play a role in type 1 diabetes. A persistent (or possibly recurrent) enterovirus infection is also consistent with clinical observations made in the TEDDY and DiViD studies. In this regard, enteroviruses with 5′ terminal genomic deletions have been detected in human heart tissue and are replication-defective [36], and this mechanism is worthy of additional attention as a plausible means by which persistence might be maintained. This question remains challenging to address because any virus present in nPOD pancreases has not proven readily cultivatable using the normal amplification methods and cell lines typically employed for wild-type CVB or echoviruses without terminal genomic deletions. However, the nPOD-Virus Group has shown that CVB3 undergoes terminal deletion after infection of the pancreas in mice [37].

Our analysis of pancreas tissue from multiple donors provides evidence that an antiviral host response is evident in beta cells (hyperexpression of HLA-I) and is associated with enterovirus VP1 positivity [22] in the pancreas of individuals with type 1 diabetes. Such a host response is classically associated with elevated IFN-α secretion during viral infection, yet it can be induced or enhanced by other factors. HLA-I hyperexpression was never observed in donors without diabetes, even among those with beta cell VP1 positivity, suggesting that this hallmark response may be specific to those who develop type 1 diabetes. Thus, the complex biology of enterovirus infections in the pancreas and the impact of differential host responses are likely to be key determinants of the course and final outcome of enterovirus infections in pancreatic beta cells in individuals predisposed to develop type 1 diabetes. Understanding what drives these host responses remains an issue of critical importance. Of note, Knebel et al [38] showed that knockout or knockdown of an endogenous RNA-editing enzyme in beta cells triggers an IFN response, islet inflammation and beta cell dysfunction in mice. While this report suggests that an antiviral response can be initiated independent of a virus after genetic manipulation of RNA editing in a mouse model, it is unknown whether such mechanisms may occur in humans with type 1 diabetes.

Our study has several limitations. First, the inability to make more-specific inferences about the biology of the infections from the study of inert pancreas samples representing a snapshot in time obtained at a time that is not linked to clinical history or infection but rather results from circumstances resulting in death that are outside of our control. As such, the plausible initial phases of infection may be challenging to identify and link to diabetes manifesting much later. Second, we could only examine a limited number of organ donors with AAbs, which are rare and difficult to identify in the general organ donor population, limiting our statistical power. Yet, this study presents the largest dataset to date, which remains extremely valuable. Third, due to sample availability and logistical considerations, not all assays could be performed on all donors. As no clear sex bias is reported in type 1 diabetes, and we were limited to the donors available within the biobank, a sex analysis was not performed.

In summary, the integrated analysis of the primary data generated by the nPOD-Virus Group shows the presence of multiple markers of enterovirus infection with a high degree of reproducibility across multiple independent laboratories studying the same donors. Critically, our approach relied on combining enterovirus-targeted and unbiased assays capable of specifically identifying multiple viruses in human pancreatic tissue; we identified both enterovirus proteins and RNA sequences by several methods (noting that when compared with RT-PCR, RNA-seq approaches may not be sensitive enough [21, 39]). Our approach to examine several markers revealed that multiple indices of viral infection were more prevalent in T1D-ICI donors and in donors with multiple AAbs compared with control donors without diabetes, donors positive for a single AAb^+^ and T1D-IDI donors. The association between markers of enterovirus infection and the presence of residual beta cells links these infections to the progression of type 1 diabetes and supports a contribution of enteroviruses to islet autoimmunity and beta cell loss, either directly or indirectly, that may continue even several years after initial disease diagnosis. We detected enterovirus RNA (confirmed by sequencing) at high frequency among AAb^+^ donors who had not yet progressed to clinical disease, which is consistent with the additional observation that 66.7% of AAb^++^ donors tested positive for enterovirus RNA when probed in situ with enterovirus-specific oligonucleotides [23]. This suggests that an active phase of enterovirus infection may occur during the early stages of the disease process. From the collective of these observations demonstrating the presence of enterovirus markers and of a host response years beyond diagnosis of diabetes, and concurrent with previous literature, we suggest that the overall picture is consistent with low-grade infections that may persist chronically, or perhaps multiple infections may occur over time. Overall, our data support a role for beta cell enterovirus infection in type 1 diabetes pathogenesis in, at least, a proportion of individuals. As such, our findings provide further support for the therapeutic rationale to intervene in disease progression by targeting viral infection, either with vaccination or with antiviral therapies [27]: a multivalent enterovirus vaccine has been recently produced [40] and, as noted above, therapy with antiviral agents was associated with preservation of insulin secretion in newly diagnosed individuals [29]. Our collective results from the analysis of pancreas and other tissues support a role for enteroviruses in type 1 diabetes and provide strong rationale for those efforts.

## Supplementary Information

Below is the link to the electronic supplementary material.

ESM (PDF 1.31 MB)

## Data Availability

Data generated and analysed during this study are available through the corresponding author upon request.

## Abbreviations

AAb: Autoantibody
CVB: Coxsackievirus B
DiViD: Diabetes Virus Detection
EADB: Exeter Archival Diabetes Biobank
EV-PCR: Enterovirus-specific RT-PCR
IHC: Immunohistochemistry
ICI: Insulin-containing islet
IDI: Insulin-deficient islet
ND: Non-diabetic
nPOD: Network for Pancreatic Organ Donors with Diabetes
smFISH: Single-molecule-based fluorescent in situ hybridisation
T1D-ICI: Type 1 diabetes with insulin-containing islets (group)
T1D-IDI: Type 1 diabetes with insulin-deficient islets (group)
VP1: Viral capsid protein 1
