# Supplementary material for: Joint analysis of the nPOD-Virus Group data: the association of enterovirus with type 1 diabetes is supported by multiple markers of infection in pancreas tissue
Source: Diabetologia. 2025 Mar 17;68(6):1226–41. doi: 10.1007/s00125-025-06401-x (PMC12069141; doi:10.1007/s00125-025-06401-x)
Supplement: Supplementary file 1 — ESM (PDF 1.31 MB) [file 125_2025_6401_MOESM1_ESM.pdf]

ESM Table 1: Donor Demographics

Study Number	RRiD	Donor Type	Age	Sex	BMI (kg/m2)	C-peptide (nmol/l)*	Duration	AAb	Presence of Insulitis	Race
6001	SAMN15879058	ND	22	Male	21.9	0.52	NA	NA	N	White
6003	SAMN15879060	ND	23	Female	29.3		NA	NA	N	White
6004	SAMN15879061	ND	33	Male	30.9		NA	NA	N	White
6005	SAMN15879062	ND	5	Female	15.7		NA	NA	N	White
6007	SAMN15879064	ND	9	Male	20		NA	NA	N	African Am
6008	SAMN15879065	ND	50	Female	24.2		NA	NA	N	White
6009	SAMN15879066	ND	45	Male	30.6	3.74	NA	NA	N	White
6010	SAMN15879067	ND	47	Female	19.7		NA	NA	N	White
6011	SAMN15879068	ND	46	Female	26.3		NA	NA	N	African Am
6012	SAMN15879069	ND	68	Female	23.7	0.98	NA	NA	N	White
6013	SAMN15879070	ND	65	Male	24.2	0.92	NA	NA	N	White
6014	SAMN15879071	ND	2	Male	20.7		NA	NA	N	White
6015	SAMN15879072	ND	39	Female	32.2	0.66	NA	NA	N	White
6016	SAMN15879073	ND	64	Female	31.2		NA	NA	N	White
6017	SAMN15879074	ND	59	Female	24.8	3.26	NA	NA	N	White
6019	SAMN15879076	ND	42	Male	31	0.16	NA	NA	N	White
6020	SAMN15879077	ND	60	Male	29.8	0.93	NA	NA	N	White
6021	SAMN15879078	ND	72	Female	24.5	7.56	NA	NA	N	Hispanic/Latino
6022	SAMN15879079	ND	75	Male	30.6	1.65	NA	NA	N	White
6024	SAMN15879081	ND	21	Male	27.8	1.16	NA	NA	N	White
6029	SAMN15879086	ND	24	Female	22.6		NA	NA	N	Hispanic/Latino
6030	SAMN15879087	ND	30.1	Male	27.1	0.84	NA	NA	N	White
6034	SAMN15879091	ND	32	Female	25.2	1.04	NA	NA	N	White
6047	SAMN15879104	ND	7.8	Male	23.9	0.21	NA	NA	N	White
6048	SAMN15879105	ND	30	Male	20.6	5.91	NA	NA	N	White
6055	SAMN15879112	ND	27	Male	22.7	0.19	NA	NA	N	White
6060	SAMN15879117	ND	24	Male	32.7	4.50	NA	NA	N	White
6073	SAMN15879130	ND	19.2	Male	36	0.23	NA	NA	N	White
6075	SAMN15879132	ND	16	Male	14.9	0.97	NA	NA	N	African Am
6091	SAMN15879148	ND	27.1	Male	35.6	2.54	NA	NA	N	White
6095	SAMN15879152	ND	40	Male	35.5		NA	NA	N	Hispanic/Latino
6096	SAMN15879153	ND	16	Female	18.8	0.98	NA	NA	N	African Am
6097	SAMN15879154	ND	43.1	Female	36.4	5.53	NA	NA	N	White
6098	SAMN15879155	ND	17.8	Male	22.8	0.47	NA	NA	N	White

6099	SAMN15879156	ND	14.2	Male	30	1.77	NA	NA	N	White
6102	SAMN15879159	ND	45.1	Female	35.1	0.18	NA	NA	N	White
6103	SAMN15879160	ND	1.5	Male	16.8	0.32	NA	NA	N	White
6104	SAMN15879161	ND	41	Male	20.5	6.78	NA	NA	N	White
6106	SAMN15879163	ND	2.9	Male	17.4	2.43	NA	NA	N	White
6112	SAMN15879169	ND	6.3	Female	18.4	1.69	NA	NA	N	Hispanic/Latino
6117	SAMN15879174	ND	0.33	Male	18.4	1.08	NA	NA	N	White
6126	SAMN15879183	ND	25.2	Male	25.1	0.29	NA	NA	N	Hispanic/Latino
6130	SAMN15879187	ND	5.2	Male	18.5	1.58	NA	NA	N	White
6131	SAMN15879188	ND	24.2	Male	24.8	0.33	NA	NA	N	White
6134	SAMN15879191	ND	26.7	Male	20.1	1.18	NA	NA	N	White
6137	SAMN15879194	ND	8.9	Female	24.2	4.00	NA	NA	N	Hispanic/Latino
6140	SAMN15879197	ND	38	Male	21.7	3.66	NA	NA	N	White
6153	SAMN15879209	ND	15.2	Male	20.5	2.77	NA	NA	N	Hispanic/Latino
6160	SAMN15879216	ND	22.1	Male	23.9	0.13	NA	NA	N	White
6162	SAMN15879218	ND	22.7	Male	28.9	2.51	NA	NA	N	African Am
6165	SAMN15879221	ND	45.8	Female	25	1.47	NA	NA	N	Caucasian
6168	SAMN15879224	ND	51	Male	25.2		NA	NA	N	Hispanic/Latino
6172	SAMN15879228	ND	19.2	Female	32.4	2.65	NA	NA	N	White
6174	SAMN15879230	ND	20.9	Male	19.5	0.99	NA	NA	N	White
6178	SAMN15879234	ND	24.5	Female	27.5	1.50	NA	NA	N	White
6179	SAMN15879235	ND	20	Female	20.7	0.90	NA	NA	N	White
6182	SAMN15879238	ND	2.7	Male	26	0.75	NA	NA	N	White
6227	SAMN15879283	ND	17	Female	26.4	0.91	NA	NA	N	White
6229	SAMN15879285	ND	31	Female	26.9	2.06	NA	NA	N	White
6232	SAMN15879288	ND	14	Female	20.8	6.44	NA	NA	N	White
6234	SAMN15879290	ND	20	Female	25.6	2.27	NA	NA	N	White
6238	SAMN15879294	ND	20	Male	21.7	0.39	NA	NA	N	African Am
6254	SAMN15879310	ND	38	Male	30.5	2.12	NA	NA	N	White
6271	SAMN15879325	ND	17	Male	24.4	3.79	NA	NA	N	White
6278	SAMN15879332	ND	12	Female	21.3	1.50	NA	NA	N	African Am
6279	SAMN15879333	ND	19	Male	34	2.64	NA	NA	N	White
6282	SAMN15879336	ND	37	Male	41.9	2.25	NA	NA	N	White
6289	SAMN15879343	ND	19	Male	38.3	2.66	NA	NA	N	African Am
6292	SAMN15879346	ND	3	Male	19.33	1.24	NA	NA	N	White
6318	SAMN15879372	ND	10	Female	17.6	1.28	NA	NA	N	White
6331	SAMN15879385	ND	27.1	Female	24	0.99	NA	NA	N	African Am

6335	SAMN15879389	ND	18.8	Male	23.6	2.92	NA	NA	N	Multiracial
6339	SAMN15879393	ND	23.2	Male	25	3.48	NA	NA	N	White
6366	SAMN15879419	ND	21	Female	20.5	0.14	NA	NA	N	Hispanic/Latino
6368	SAMN15879421	ND	38.3	Male	20.7	1.01	NA	NA	N	White
6375	SAMN15879428	ND	28.7	Male	31.8	5.72	NA	NA	N	White
6384	SAMN15879437	ND	17	Male	18.2	0.23	NA	NA	N	White
6386	SAMN15879439	ND	14	Male	23.9	0.37	NA	NA	N	White
6389	SAMN15879442	ND	18.6	Male	20.9	2.38	NA	NA	N	White
6401	SAMN15879454	ND	25.07	Female	31.3	4.23	NA	NA	N	Hispanic/Latino
6406	SAMN15879459	ND	6.9	Male	16.8	1.34	NA	NA	N	White
6413	SAMN15879466	ND	10.1	Female	19	1.74	NA	NA	N	White
6482	SAMN15879535	ND	18.69	Female	20	2.47	NA	NA	N	White
6002	SAMN15879059	AAb+	39	Male	23.7		NA	mIAA+	N	White
6023	SAMN15879080	AAb+	66	Male	34		NA	mIAA+	N	White
6027	SAMN15879084	AAb+	18.8	Male	19.9		NA	ZnT8A+	N	White
6044	SAMN15879101	AAb+	41.4	Male	27.4	4.47	NA	GADA+	N	Hispanic/Latino
6090	SAMN15879147	AAb+	2.2	Male	18.8	1.76	NA	GADA+	N	Hispanic/Latino
6101	SAMN15879158	AAb+	64.8	Male	34.3	8.64	NA	GADA+	N	White
6116	SAMN15879173	AAb+	0.17	Female	23.6	0.33	NA	mIAA+	N	Hispanic/Latino
6123	SAMN15879180	AAb+	23.2	Female	17.6	0.66	NA	GADA+	N	White
6147	SAMN15879203	AAb+	23.8	Female	32.9	1.05	NA	GADA+	N	White
6151	SAMN15879207	AAb+	30	Male	24.2	1.81	NA	GADA+	N	White
6154	SAMN15879210	AAb+	48.5	Female	24.5	0.02	NA	GADA+	N	White
6156	SAMN15879212	AAb+	40	Male	19.8	4.40	NA	GADA+	N	White
6171	SAMN15879227	AAb+	4.4	Female	14.8	2.95	NA	GADA+	N	White
6181	SAMN15879237	AAb+	31.9	Male	21.9	0.02	NA	GADA+	N	White
6184	SAMN15879240	AAb+	47.6	Female	27	1.13	NA	GADA+	N	Hispanic/Latino
6301	SAMN15879355	AAb+	26	Male	32.1	1.29	NA	GADA+	N	African Am
6303	SAMN15879357	AAb+	22	Male	31.9	1.00	NA	GADA+	N	White
6310	SAMN15879364	AAb+	28	Female	22.4	3.48	NA	GADA+	Y	Hispanic/Latino
6314	SAMN15879368	AAb+	21	Male	23.8	0.49	NA	GADA+	N	White
6397	SAMN15879450	AAb+	21.16	Female	29.6	4.21	NA	GADA+	N	White
6400	SAMN15879453	AAb+	25.15	Male	22.2	1.38	NA	GADA+	N	Hispanic/Latino
6421	SAMN15879474	AAb+	6.73	Male	17.9	0.61	NA	GADA+	N	Hispanic/Latino
6080	SAMN15879137	AAb++	69.2	Female	21.3	0.61	NA	mIAA+ GADA+	N	White
6158	SAMN15879214	AAb++	40.3	Male	29.7	0.17	NA	mIAA+ GADA+	N	White
6167	SAMN15879223	AAb++	37	Male	26.3	1.79	NA	IA2A+ ZnT8A+	N	White

6197	SAMN15879253	AAb++	22	Male	28.2	5.77	NA	GADA+ IA2A+	Y	African Am
6267	SAMN15879321	AAb++	23	Female	23.5	5.47	NA	GADA+ IA2A+	Y	White
6388	SAMN15879441	AAb++	25.2	Female	26	0.46	NA	mIAA+ GADA+	N	Hispanic/Latino
6424	SAMN15879477	AAb++	17.65	Male	51.4	2.30	NA	mIAA+ GADA+	N	Hispanic/Latino
6429	SAMN15879482	AAb++	22.1	Male	19.6	0.74	NA	mIAA+ GADA+	N	African Am
6450	SAMN15879503	AAb++	22	Female	24.4	1.81	NA	GADA+ ZnT8A+	Y	White
6038	SAMN15879095	T1D ICI	37.2	Female	30.9	0.07	20	Negative	N	White
6046	SAMN15879103	T1D ICI	18.8	Female	25.2	nd	8	IA2A+ ZnT8A+	Y	White
6049	SAMN15879106	T1D ICI	15	Female	20.8	nd	10	GADA+ mIAA+	N	African Am
6051	SAMN15879108	T1D ICI	20.3	Male	21.5	nd	13	mIAA+	N	White
6052	SAMN15879109	T1D ICI	12	Male	20.3	0.06	1	IA-2A+, mIAA+	Y	African Am
6065	SAMN15879122	T1D ICI	79	Female	29	nd	56	Negative	N	White
6070	SAMN15879127	T1D ICI	22.6	Female	21.6	nd	7	IA-2A+, mIAA+	Y	White
6084	SAMN15879141	T1D ICI	14.2	Male	26.3	nd	4	mIAA+	Y	White
6088	SAMN15879145	T1D ICI	31.2	Male	27	nd	5	GADA+ IA2A+ mIAA+ ZnT8A+	Y	White
6113	SAMN15879170	T1D ICI	13.1	Female	24.75	nd	1.58	mIAA+	Y	White
6180	SAMN15879236	T1D ICI	27.1	Male	25.9	nd	11	GADA+ IA2A+ mIAA+	N	White
6195	SAMN15879251	T1D ICI	19.3	Male	23.7	nd	5	GADA+ IA2A+ mIAA+ ZnT8A+	Y	White
6196	SAMN15879252	T1D ICI	26.5	Female	26.6	0.16	15	GADA+ mIAA+	N	African Am
6198	SAMN15879254	T1D ICI	22	Female	23.1	nd	3	GADA+ IA2A+ mIAA+ ZnT8A+	Y	Hispanic/Latino
6209	SAMN15879265	T1D ICI	5	Female	15.9	0.03	0.25	IA2A+ mIAA+ ZnT8A+	Y	White
6211	SAMN15879267	T1D ICI	24	Female	24.4	nd	4	GADA+ IA2A+ mIAA+ ZnT8A+	Y	African Am
6212	SAMN15879268	T1D ICI	20	Male	29.1	nd	5	mIAA+	Y	White
6228	SAMN15879284	T1D ICI	13	Male	17.4	0.03	0	GADA+ IA2A+ ZnT8A+	Y	White
6243	SAMN15879299	T1D ICI	13	Male	21.3	0.14	5	mIAA+	Y	White
6245	SAMN15879301	T1D ICI	22	Male	23.2	nd	7	GADA+ IA2A+	Y	White
6247	SAMN15879303	T1D ICI	24	Male	24.3	0.16	0.6	mIAA+	Y	White
6264	SAMN15879318	T1D ICI	12	Female	22	nd	9	Negative	Y	White
6265	SAMN15879319	T1D ICI	11	Male	12.9	0.02	8	GADA+ mIAA+	Y	White
6302	SAMN15879356	T1D ICI	38.5	Male	20.5	0.06	32.5	Negative	N	African Am
6306	SAMN15879360	T1D ICI	19	Male	24.5	nd	5	mIAA+	Y	White
6307	SAMN15879361	T1D ICI	45	Female	19.5	nd	10	GADA+ mIAA+	N	White
6323	SAMN15879377	T1D ICI	22	Female	24.7	nd	6	GADA+ IA2A+	Y	White
6325	SAMN15879379	T1D ICI	20	Female	31.2	0.05	6	GADA+ IA2A+ mIAA+	Y	African Am
6328	SAMN15879382	T1D ICI	39	Male	24	nd	20	GADA+ mIAA+	Y	Hispanic/Latino
6337	SAMN15879391	T1D ICI	20.6	Female	17.9	nd	5	mIAA+	N	White
6342	SAMN15879396	T1D ICI	14	Female	24.3	0.09	2	IA2A+ mIAA+	Y	White

6362	SAMN15879415	T1D ICI	24.9	Male	28.5	0.13	0	GADA+	Y	White
6367	SAMN15879420	T1D ICI	24	Male	25.7	0.13	2	Negative	N	White
6371	SAMN15879424	T1D ICI	12.5	Female	16.6	0.04	2	GADA+ IA2A+ mIAA+ ZnT8A+	Y	White
6380	SAMN15879433	T1D ICI	11.6	Female	14.6	0.07	0	Negative	Y	African Am
6396	SAMN15879449	T1D ICI	17.1	Female	22.6	0.02	2	Negative	Y	White
6405	SAMN15879458	T1D ICI	29.1	Female	42.5	0.61	0.6	GADA+ IA2A+ ZnT8A+	Y	Hispanic/Latino
6414	SAMN15879467	T1D ICI	23.1	Male	28.4	0.05	0.43	GADA+ mIAA+ ZnT8A+	Y	African Am
6449	SAMN15879502	T1D ICI	24	Male	23.02	0.01	2	IA2A+ mIAA+ ZnT8A+	Y	White
6456	SAMN15879509	T1D ICI	30.49	Female	30.1	3.41	0	GADA+ ZnT8A+	Y	African Am
6469	SAMN15879522	T1D ICI	27.06	Female	26.9	0.22	1.5	GADA+	Y	White
6025	SAMN15879082	T1D IDI	23.8	Male	26.6	nd	19	mIAA+	N	White
6026	SAMN15879083	T1D IDI	22.4	Male	24.1	nd	9	mIAA+	N	White
6031	SAMN15879088	T1D IDI	39	Male	24.5	nd	35	mIAA+	N	White
6032	SAMN15879089	T1D IDI	33.8	Male	29.4	nd		mIAA+	N	White
6035	SAMN15879092	T1D IDI	32.1	Male	27.1	nd	28	mIAA+	N	White
6039	SAMN15879096	T1D IDI	28.7	Female	23.4	nd	12	GADA+ IA2A+ mIAA+ ZnT8A+	Y	White
6040	SAMN15879097	T1D IDI	50	Female	31.6	nd	20	mIAA+	N	White
6041	SAMN15879098	T1D IDI	26.3	Male	28.4	nd	23	Negative	N	White
6045	SAMN15879102	T1D IDI	26.4	Male	23.1	nd	8	mIAA+ ZnT8A+	N	White
6063	SAMN15879120	T1D IDI	4.4	Male	23.8	nd	3	mIAA+	N	White
6064	SAMN15879121	T1D IDI	22.6	Female	19.6	nd	9	GADA+ IA2A+ mIAA+ ZnT8A+	N	White
6066	SAMN15879123	T1D IDI	78	Male	30.9	nd	74	IA2A+ mIAA+	N	White
6067	SAMN15879124	T1D IDI	32.6	Female	26.8	nd	8	Negative	N	Hispanic/Latino
6068	SAMN15879125	T1D IDI	72	Female	21.9	nd	69	GADA+	N	White
6076	SAMN15879133	T1D IDI	25.8	Male	18.8	nd	15	GADA+ mIAA+	N	White
6077	SAMN15879134	T1D IDI	32.9	Female	22	nd	19	mIAA+	N	White
6078	SAMN15879135	T1D IDI	59	Male	21.6	nd	52	Negative	N	White
6079	SAMN15879136	T1D IDI	11.1	Female	18.6	nd	8	Negative	N	White
6083	SAMN15879140	T1D IDI	15.2	Female	18.4	nd	11	mIAA+	N	White
6086	SAMN15879143	T1D IDI	71	Female	23.6	nd	63	Negative	N	American Indian/Alaska Native
6087	SAMN15879144	T1D IDI	17.5	Male	21.9	nd	4	mIAA+ ZnT8A+	N	White
6089	SAMN15879146	T1D IDI	14.3	Male	26	nd	8	mIAA+	N	White
6119	SAMN15879176	T1D IDI	7.8	Male	19.4	nd	14	GADA+ mIAA+	N	White
6128	SAMN15879185	T1D IDI	33.8	Female	22.2	nd	31.5	mIAA+	N	White
6135	SAMN15879192	T1D IDI	43.5	Male	28.7	nd	21	GADA+ mIAA+	N	White
6138	SAMN15879195	T1D IDI	49.2	Female	33.7	nd	41	mIAA+	N	White
6141	SAMN15879198	T1D IDI	36.7	Male	26	nd	28	GADA+ IA2A+ mIAA+ ZnT8A+	N	White

6143	SAMN15879200	T1D IDI	32.6	Female	26.1	nd	7	IA2A+ mIAA+	N	White
6145	SAMN15879202	T1D IDI	18	Male	23.1	0.02	11	GADA+ mIAA+ ZnT8A+	N	White
6148	SAMN15879204	T1D IDI	17.1	Male	23.9	nd	7	GADA+ mIAA+	N	White
6152	SAMN15879208	T1D IDI	29.6	Female	30.1	nd	12	ZnT8A+	N	White
6155	SAMN15879211	T1D IDI	50	Female	26	nd	43	mIAA+	N	White
6159	SAMN15879215	T1D IDI	50.8	Female	35.5	nd	44	mIAA+	N	White
6161	SAMN15879217	T1D IDI	19.2	Female	36.1	nd	7	IA2A+ mIAA+	N	White
6163	SAMN15879219	T1D IDI	32.5	Male	25.5	nd	30	IA2A+ mIAA+	N	White
6169	SAMN15879225	T1D IDI	27.6	Female	25	nd	15	GADA+ mIAA+	N	Hispanic/Latino
6173	SAMN15879229	T1D IDI	44.1	Male	23.9	nd	15	Negative	N	White
6205	SAMN15879261	T1D ICI	40.9	Female	22.6	0.05	33	mIAA+	N	White
6207	SAMN15879263	T1D IDI	16.7	Female	24.4	nd	10	IA2A+ mIAA+ ZnT8A+	N	African Am
6208	SAMN15879264	T1D IDI	32.6	Female	23.4	nd	16	Negative	N	White
6224	SAMN15879280	T1D IDI	21	Female	22.8	nd	1.5	Negative	N	White
6324	SAMN15879378	T1D IDI	29	Male	26.2	nd	2	GADA+ mIAA+	Y	Hispanic/Latino

* nd - not detectable; NA - not available; RRiD - Research Resource Identifiers, https://www.rrids.org/

ESM Table 2: Pairwise combinations of VP1, proteomics, EV-PCR and HLA-I assays. Fishers Exact Test Two-sided comparing outputs from the ND v T1D-ICI assays. Total donors assess [number positive].

Assays compared	ND<br>No. Donors<br>[No. Double positive]	T1D ICI<br>No. Donors<br>[No. Double positive]	p value
**VP1 HLA-I**	54 [0]	38 [28]	**<0.0001**
**VP1 EV-PCR**	49 [1]	32 [4]	0.07
**VP1 Proteomics**	24 [5]	22 [11]	0.06
**EV-PCR HLAI**	36 [0]	30 [5]	**0.016**
**EV-PCR Proteomics**	20 [0]	22 [3]	0.61
**Proteomics HLA-I**	24 [0]	23 [14]	**<0.0001**

ESM Table 3: Pairwise combinations of smFISH analysis with VP1, proteomics, EV-PCR and HLA-I. Fishers Exact Test Two-sided comparing outputs from the ND v T1D-ICI assays. Total donors assess [number positive].

Assays compared	ND<br>No. Donors<br>[No. Double positive]	T1D ICI<br>No. Donors<br>[No. Double positive]	p value
**VP1 smFISH**	14 [0]	10 [6]	**0.0016**
**Proteomics smFISH**	11 [0]	10 [4]	**0.0351**
**EV-PCR smFISH**	14 [0]	9 [2]	0.1423
**HLA-I smFISH**	14 [0]	11 [7]	**0.0007**

ESM Table 4: Combination of positive markers of viral infection in donors with evidence of autoimmunity, or autoimmunity and beta cells. The total number of donors assayed in each group are shown with the number of donors positive for ≥2 assays and percentage positive. Type 1 diabetes and residual insulin containing islets (T1D-ICI), Type 1 diabetes and only insulin deficient islets (T1D-IDI); no diabetes (ND); single (AAb+) or multiple AAb (AAb++) autoantibodies.

**Donor Type**	**Total donors**	**≥2 positive**	**%**
**Donors with autoimmunity**	59	28	47.46
AAb+/++	22	6	27.27
T1D-ICI	26	22	84.62
T1D-IDI	11	0	0.00
	**Total donors**	**≥2 positive**	**%**
**Donors with autoimmunity and beta cells**	48	28	58.33
AAb+/++	22	6	27.27
T1D-ICI	26	22	84.62
	**Total donors**	**≥2 positive**	**%**
**ND**	36	0	0

ESM Table 5: Agreement Analysis assessing concordance between pairs of assays.

Donor Type	Comparison	n	Agreement	Agreement Coefficient	Agreement Coefficient 95% CL	Agreement p-value	overall agreement	% negative agreement (95 CL)	% positive agreement (95 CL)
	HLA vs. RNAseq	20	Almost perfect	1	(1,1)	0.0000	100%	100(100,100)	-
	smFISH vs. HLA	14	Almost perfect	1	(1,1)	0.0000	100%	100(100,100)	
	smFISH vs. RNAseq	5	Almost perfect	1	(1,1)	0.0000	100%	100(100,100)	
	HLA vs. EV PCR	36	Almost perfect	0.94127	(0.854,1)	0.0000	94%	97(93,100)	-
	smFISH vs. EV PCR	14	Almost perfect	0.92329	(0.746,1)	0.0000	93%	96(89,100)	
	EV PCR vs. RNAseq	22	Almost perfect	0.90045	(0.745,1)	0.0000	91%	95(89,100)	-
	VP1 vs. RNAseq	23	Moderate	0.58981	(0.237,0.942)	0.0011	70%	82(69,95)	-
	All Assays (if RNAseq ignored)	11	Moderate	0.48926	(0.161,0.817)	0.0039	65%	-	-
ND	Proteomics vs. RNAseq	11	Moderate	0.48235	(-0.159,1)	0.0624	64%	78(57,99)	-
	All Assays	4	Moderate	0.47772	(-0.349,1)	0.0817	65%	-	-
	EV PCR vs. Proteomics	20	Moderate	0.46257	(0.013,0.912)	0.0221	65%	77(61,94)	22(0,58)
	HLA vs. Proteomics	24	Fair	0.37824	(-0.054,0.811)	0.0417	58%	74(58,89)	-
	VP1 vs. HLA	54	Fair	0.3593	(0.079,0.64)	0.0065	57%	73(62,84)	-
	smFISH vs. VP1	14	Fair	0.35385	(-0.258,0.966)	0.1169	57%	73(52,94)	
	smFISH vs. Proteomics	11	Fair	0.29936	(-0.448,1)	0.1966	55%	71(46,95)	
	VP1 vs. EV PCR	49	Fair	0.24286	(-0.074,0.56)	0.0652	53%	68(56,81)	8(0,23)
	VP1 vs. Proteomics	24	Slight	0.18919	(-0.249,0.627)	0.1903	58%	64(44,85)	50(23,77)

Donor Type	Comparison	n	Agreement	Agreement Coefficient	Agreement Coefficient 95% CL	Agreement p-value	overall agreement	% negative agreement (95 CL)	% positive agreement (95 CL)
	Proteomics vs. RNAseq	8	Almost perfect	0.85841	(0.482,1)	0.0005	88%	93(80,100)	-
	HLA vs. RNAseq	16	Substantial	0.77412	(0.468,1)	0.0000	81%	90(78,100)	-
	VP1 vs. HLA	28	Substantial	0.63793	(0.331,0.945)	0.0001	79%	85(73,97)	63(35,90)
	EV PCR vs. Proteomics	13	Moderate	0.52294	(-0.033,1)	0.0314	69%	80(61,99)	33(-15,82)
	HLA vs. Proteomics	15	Moderate	0.52	(0.015,1)	0.0223	73%	80(61,99)	60(24,96)
	VP1 vs. RNAseq	16	Moderate	0.46067	(-0.052,0.973)	0.0373	63%	77(59,95)	-
	smFISH vs. HLA	7	Fair	0.20755	(-0.846,1)	0.3235	57%	67(31,100)	
	smFISH vs. EV PCR	4	Slight	0.2	(-1.858,1)	0.3887	50%	67(23,100)	
AAb+/++	EV PCR vs. RNAseq	16	Slight	0.2	(-0.416,0.816)	0.2499	50%	67(45,88)	-
	VP1 vs. EV PCR	22	Slight	0.15385	(-0.332,0.639)	0.2585	55%	64(44,85)	38(7,68)
	VP1 vs. Proteomics	14	Slight	0.0439	(-0.597,0.685)	0.4423	50%	59(31,87)	36(0,73)
	HLA vs. EV PCR	22	Poor	0.0365	(-0.487,0.56)	0.4431	45%	60(39,81)	14(0,39)
	All Assays (if RNAseq ignored)	3	Poor	-0.06195	(-0.795,0.671)	0.6245	47%	-	-
	smFISH vs. VP1	6	Poor	-0.2	(-1.566,1)	0.6390	33%	50(8,92)	
	smFISH vs. Proteomics	7	Poor	-0.68	(-1.53,0.17)	0.9511	14%		23(-15,65)
	smFISH vs. RNAseq	0		-	-	-	-	-	-
	All Assays	0		-	-	-	-	-	-

Donor Type	Comparison	n	Agreement	Agreement Coefficient	Agreement Coefficient 95% CL	Agreement p-value	overall agreement	% negative agreement (95 CL)	% positive agreement (95 CL)
	VP1 vs. HLA	38	Substantial	0.68523	(0.456,0.915)	0.0000	76%	18(-12,49)	86(77,95)
	EV PCR vs. RNAseq	15	Moderate	0.53846	(0.049,1)	0.0166	67%	80(63,97)	-
	smFISH vs. VP1	10	Moderate	0.52	(-0.149,1)	0.0564	70%	40(-14,94)	80(58,100)
	smFISH vs. HLA	11	Moderate	0.48235	(-0.159,1)	0.0624	64%		78(57,99)
	HLA vs. Proteomics	23	Moderate	0.42897	(0.005,0.853)	0.0239	61%	-	76(60,91)
	VP1 vs. Proteomics	22	Slight	0.2	(-0.308,0.708)	0.2112	50%	-	67(48,85)
	smFISH vs. RNAseq	4	Slight	0.2	(-1.858,1)	0.3887	50%	67(23,100)	
	All Assays	4	Slight	0.1	(-0.457,0.657)	0.3038	50%	-	-
T1D ICI	All Assays (if RNAseq ignored)	9	Slight	0.08828	(-0.153,0.329)	0.2115	49%	-	-
	smFISH vs. Proteomics	10	Slight	0.08257	(-0.752,0.917)	0.4139	50%	29(-15,72)	62(30,93)
	Proteomics vs. RNAseq	14	Slight	0.03448	(-0.674,0.743)	0.4589	43%	60(35,85)	-
	EV PCR vs. Proteomics	22	Poor	-0.16024	(-0.629,0.309)	0.7574	41%	48(24,72)	32(4,59)
	VP1 vs. EV PCR	33	Poor	-0.38884	(-0.726,-0.052)	0.9875	30%	34(14,55)	26(5,46)
	VP1 vs. RNAseq	16	Poor	-0.41176	(-1.019,0.195)	0.9156	25%	40(13,67)	-
	smFISH vs. EV PCR	9	Poor	-0.55556	(-1.233,0.122)	0.9523	22%	22(-14,58)	22(-14,58)
	HLA vs. EV PCR	30	Poor	-0.62162	(-0.971,-0.272)	0.9995	17%	-	29(9,48)
	HLA vs. RNAseq	16	Poor	-1	(-1,-1)	1.0000	0%	-	-

Donor Type	Comparison	n	Agreement	Agreement Coefficient	Agreement Coefficient 95% CL	Agreement p-value	overall agreement	% negative agreement (95 CL)	% positive agreement (95 CL)
	EV PCR vs. RNAseq	10	Almost perfect	1	(1,1)	0.0000	100%	100(100,100)	-
	HLA vs. EV PCR	22	Almost perfect	0.90045	(0.745,1)	0.0000	91%	95(89,100)	-
	VP1 vs. RNAseq	10	Substantial	0.7561	(0.316,1)	0.0018	80%	89(74,100)	-
	HLA vs. RNAseq	10	Substantial	0.7561	(0.316,1)	0.0018	80%	89(74,100)	-
	VP1 vs. EV PCR	33	Moderate	0.53846	(0.231,0.846)	0.0006	67%	80(68,92)	-
	VP1 vs. HLA	27	Fair	0.32075	(-0.101,0.742)	0.0650	56%	71(55,87)	-
	VP1 vs. Proteomics	2		-	-	-	-	-	-
	EV PCR vs. Proteomics	2		-	-	-	-	-	-
T1D IDI	HLA vs. Proteomics	2		-	-	-	-	-	-
	Proteomics vs. RNAseq	2		-	-	-	-	-	-
	smFISH vs. EV PCR	1		-	-	-	-	-	-
	smFISH vs. Proteomics	0		-	-	-	-	-	-
	smFISH vs. VP1	1		-	-	-	-	-	-
	smFISH vs. HLA	1		-	-	-	-	-	-
	smFISH vs. RNAseq	1		-	-	-	-	-	-
	All Assays	2		-	-	-	-	-	-
	All Assays (if RNAseq ignored)	2		-	-	-	-	-	-

Donor Type	Comparison	n	Agreement	Agreement Coefficient	Agreement Coefficient 95% CL	Agreement p-value	overall agreement	% negative agreement (95 CL)	% positive agreement (95 CL)
	smFISH vs. RNAseq	10	Substantial	0.7561	(0.316,1)	0.0018	80%	89(74,100)	-
	EV PCR vs. RNAseq	63	Substantial	0.69871	(0.53,0.867)	0.0000	76%	86(80,93)	-
	smFISH vs. HLA	33	Substantial	0.61144	(0.322,0.9)	0.0001	79%	84(72,96)	70(48,91)
	HLA vs. RNAseq	62	Moderate	0.52869	(0.308,0.75)	0.0000	66%	80(71,88)	-
	Proteomics vs. RNAseq	35	Moderate	0.46752	(0.145,0.79)	0.0029	63%	77(65,89)	-
	smFISH vs. EV PCR	28	Moderate	0.46154	(0.092,0.831)	0.0081	64%	77(64,91)	17(0,45)
	HLA vs. EV PCR	110	Moderate	0.41234	(0.227,0.598)	0.0000	63%	75(68,82)	23(8,38)
	VP1 vs. RNAseq	65	Fair	0.38084	(0.131,0.631)	0.0017	58%	74(64,83)	-
All	VP1 vs. HLA	147	Fair	0.34869	(0.19,0.507)	0.0000	66%	72(64,79)	57(46,68)
	EV PCR vs. Proteomics	57	Fair	0.28145	(0.003,0.56)	0.0240	60%	70(59,81)	29(10,49)
	HLA vs. Proteomics	64	Fair	0.25653	(0.011,0.502)	0.0205	63%	66(53,79)	59(44,74)
	smFISH vs. VP1	31	Slight	0.19157	(-0.19,0.573)	0.1569	58%	65(47,83)	48(24,72)
	All Assays	8	Slight	0.16308	(-0.225,0.551)	0.1765	58%	-	-
	VP1 vs. EV PCR	137	Slight	0.15342	(-0.036,0.343)	0.0561	51%	66(57,73)	19(8,31)
	All Assays (if RNAseq ignored)	23	Slight	0.15123	(-0.058,0.36)	0.0736	57%	-	-
	VP1 vs. Proteomics	62	Slight	0.0667	(-0.19,0.323)	0.3025	53%	51(35,66)	55(41,70)

smFISH vs. Proteomics	28	Poor	-0.13706	(-0.533,0.259)	0.7580	43%	47(25,69)	38(15,62)

NOTES

Calculations for negative and positive agreement based on the following papers: 1) Fleiss JL. In: Statistical methods for rates and proportions. 2nded. New York: Wiley; 1981. p. 212–36, and 2) Cicchetti DV, Feinstein AR. High agreement but low kappa: IIresolving the paradoxes. J Clin Epidemiol 1990;43:551–8. In a 2 x 2 table, the formula is 2a/(2a+b+c) where a is the positive agreement, and b and c are the disagreements. This formula is used in the cases where there is no obvious gold standard (i.e. one rater or another).

ESM Fig. 1: Combinations of VP1 and smFISH; Proteomics and smFISH; EV-PCR and smFISH; and HLAI and smFISH across different donors groups reveals that donors with type 1 diabetes and residual beta cells (T1D-ICI) have an increased % of donors who are double positive (red) for the assays compared to donors without diabetes (ND). The number within each donut represents the total number of donors assessed in that donor group.

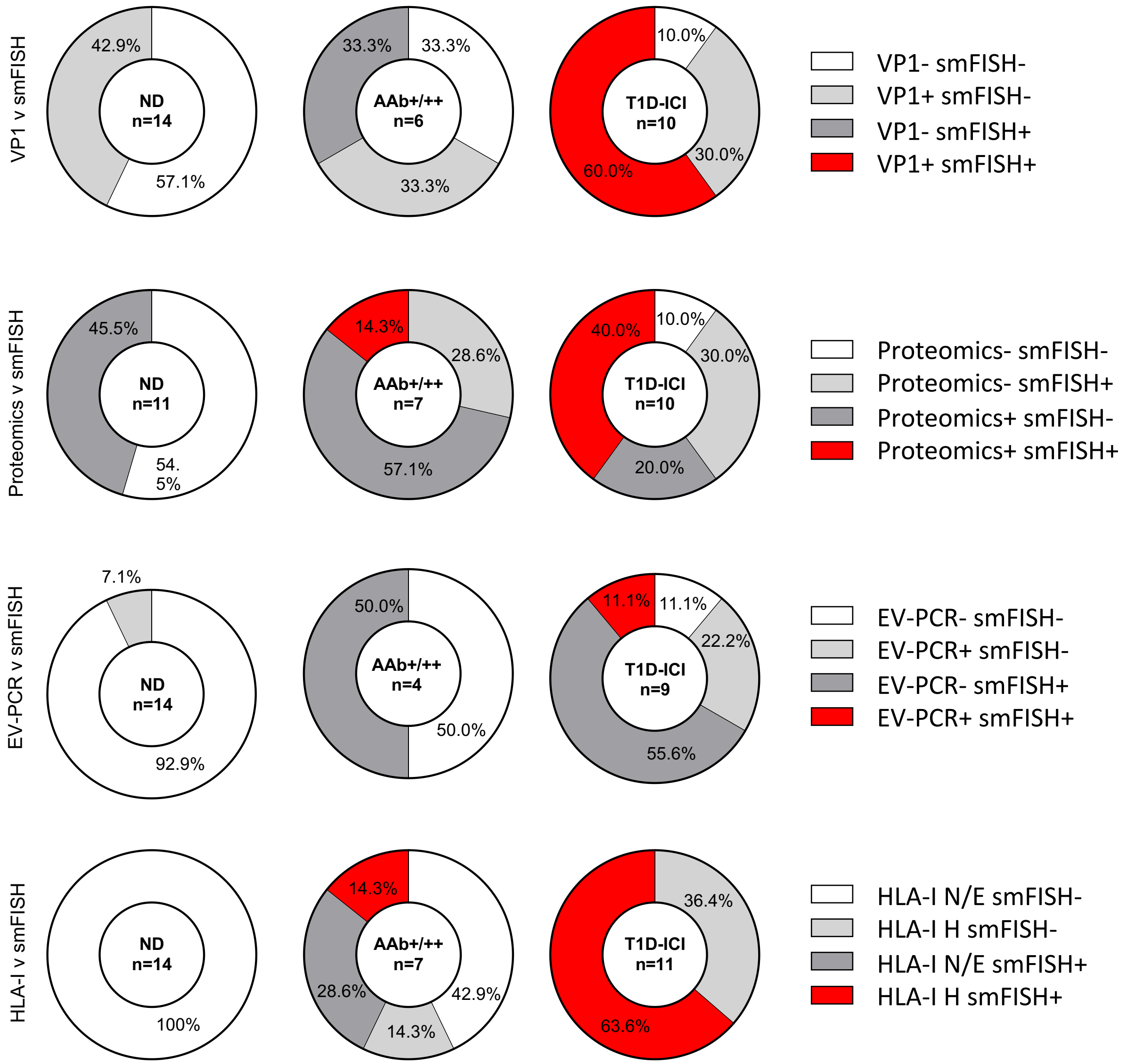

ESM Fig 2: a. Examination of donors (ND, AAb+/++ and T1D ICIs in which five assays (VP1, EV-PCR, Proteomics, HLAI and smFISH; n=23) were performed reveals that AAb+/++ donors and donors with T1D and residual ICIs are significantly more likely to have ≥ 2 assays positive when compared to donors without diabetes (ND) or evidence of islet autoimmunity (AAb). Fishers Exact Test Two sided ** p<0.01. b. Examination of extended EV-specific assay panel which includes smFISH, proteomics, EV-PCR and VP1 in ND (n=11) and T1D ICI (n=9) revealed that all T1D donors were positive for 2 or more assays, with one donor positive for all four.

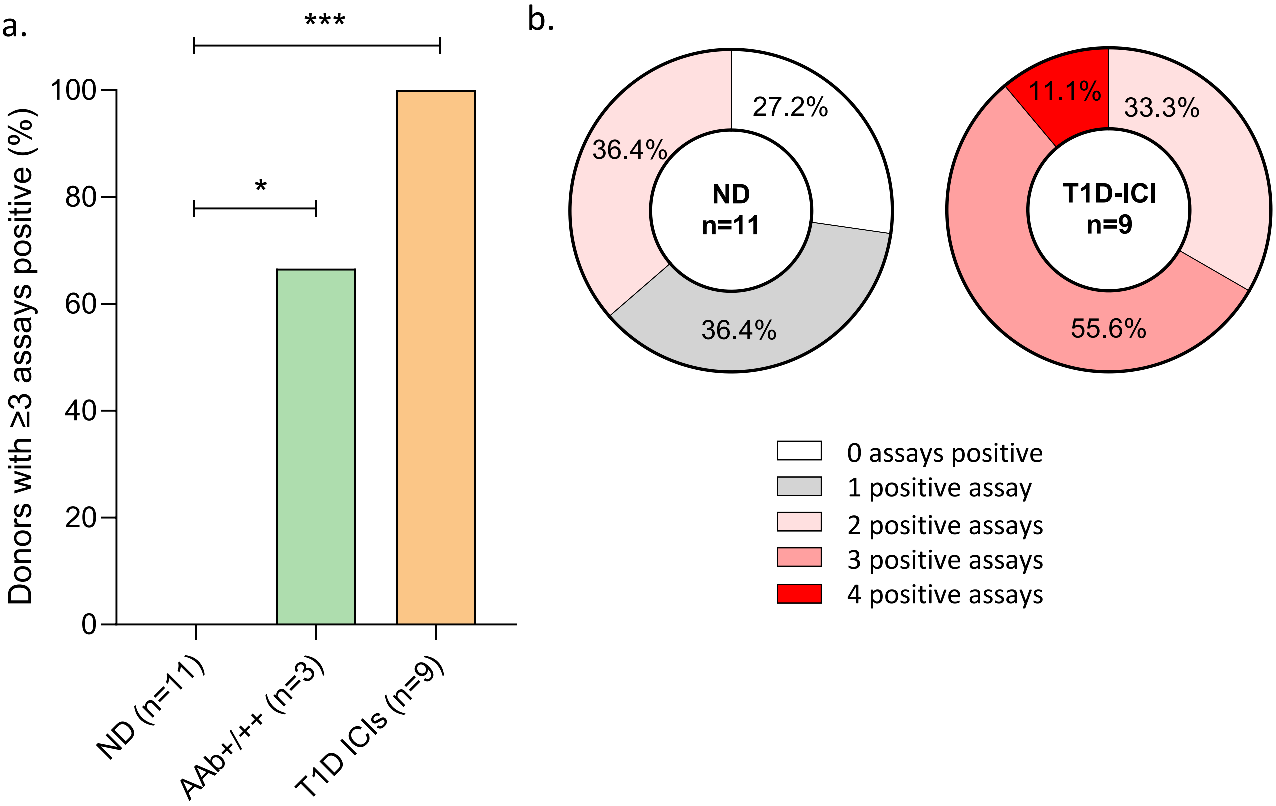

ESM Methods:

Please refer to accompanying manuscripts for more detailed information on each of the different assays (Rodriguez-Calvo et al, Laiho et al,) and Nyalwidhe et al, (https://www.medrxiv.org/content/10.1101/2024.10.24.24315944v1.full)

Immunohistochemistry and immunofluorescence for VP1 and HLA-I. We examined pancreatic sections from 188 donors, and sections were available for more than one pancreatic region from 70 donors (31 ND, 9 AAb+, 5 AAb++, 15 T1D-ICI and 10 T1D-IDI). The total number of sections per region analyzed was: head n=62, body n=74 and tail n=84. Sections were stained for insulin, glucagon, VP1 and HLA-I. Staining for VP1 was performed using the anti-enterovirus VP1 clone 5D8/1 (Agilent). In Exeter, serial FFPE sections were heated in 10 mmol/l citrate (pH 6.0) in a pressure cooker in a microwave oven at 800 W for 20 min, then cooled at room temperature for 20 min. The anti-VP1 [5D8/1] monoclonal antibody (55 ng/ml) or the HLA-I [EMR8-5] monoclonal antibody (1/1500) were incubated for 1 hour at room temperature, and the EnVision HRP Detection System (Agilent, Stockport, UK) was used for antigen detection (1-3). Serial sections were stained with anti-insulin antibody (C#A0564; Agilent, Stockport, UK; 1:600 for 1h) and visualized using the Dako REAL Envision HRP detection system. Sections were subsequently stained with anti-glucagon antibody [K79bB10] (Abcam, 1:2,000 for 1h) and visualized with the Vector AP-ABC kit combined with Vector Red substrate. All slides were dehydrated and mounted in Agilent Fluorescence Mounting medium. Sections were analyzed by brightfield microscopy using either a Nikon 50i microscope fitted with a DS-Fi camera and DSL2 camera control unit, or the sections were scanned at 40X magnification using an Akoya Biosciences Vectra® Polaris™ Automated Quantitative Pathology Imaging System. In Tampere, FFPE sections were stained with the same VP1 antibody (clone

5D8/1, sourced from DakoCytomation, Glostrup, Denmark; 1:300) using a Ventana BenchMark LT (Ventana Medical Systems, Inc.) and the ultraView™ Universal detection systems. Consecutive pancreas sections were stained using an anti-insulin antibody (Ab-6, Thermo Scientific, 1:2,000). Sections were analyzed by brightfield microscopy using either an Olympus BX60 microscope fitted with an Olympus Colorview III camera, or from scanned whole-slide images (SlideStrider scanner, Jilab Inc., Tampere, Finland). In La Jolla/Munich, pancreatic frozen sections (n= 118) were stained for insulin, glucagon and HLA-I. Tissue sections were fixed with 1% paraformaldehyde and blocked with 2% goat serum. The following primary antibodies were incubated for 1h at room temperature: Polyclonal guinea pig anti-insulin (C#A0564, Agilent, Stockport, UK; 1:140), monoclonal recombinant rabbit anti-glucagon (C#ab92517, Abcam, Cambridge, UK; 1:400) and mouse monoclonal anti human HLA-ABC (C#R7000, W6/32 clone; Agilent, Stockport, UK, 1:100). After 1 hour incubation at room temperature and washes, sections were incubated with the following secondary, fluorescently labeled, antibodies: goat anti-guinea pig IgG (H+L) Alexa Fluor 488, F(ab')2-Goat anti-Rabbit IgG (H+L) Alexa Fluor 555, and goat anti-mouse IgG2a Alexa Fluor 647 (1:1,000; all from Invitrogen, Waltham, USA). Sections were counterstained with Hoechst 33342 (C#H3570, Invitrogen, Waltham, USA, 1:5,000) for 8 min and mounted with Prolong Gold Antifade (C#P36930, Invitrogen, Waltham, USA). Sections were analyzed manually using a Nikon digital DXM1200C camera and Nikon ACT-1C Camera Controller Software or scanned by an Axio Scan.Z1 slide scanner (Zeiss, Jena, Germany) using a 20×/0.8 numerical aperture (NA) Plan-Apochromat (a = 0.55 mm) objective lens. Scanned sections were visualized using ZEN Blue 2.3 software (Zeiss, Jena, Germany). The analysis was performed by researchers blinded to the study groups.

Criteria for enterovirus and HLA-I positivity. For each donor and section, we evaluated the VP1 staining pattern in individual cells, and classified them as VP1 negative (VP1-) or VP1 positive (VP1+). A donor was considered VP1+ in the presence of ≥ 1 strongly stained VP1+ cell within any islet of a section. If multiple sections or pancreatic regions were analyzed per donor, a VP1+ cell in any of the sections/regions, from either of the different laboratories, was sufficient to define the donor as VP1+. Thus, any donor identified as VP1+ in either laboratory was recorded as VP1+, and any donor scored positive but analyzed in a single laboratory was included. We classified islets into three categories according to their HLA-I staining intensity, normal, elevated, and hyperexpression, if they had at least one islet in these categories (4).

Proteomics Methods (see Nyalwidhe et al, https://www.medrxiv.org/content/10.1101/2024.10.24.24315944v1.full

Extraction and Processing of Proteins for Mass Spectrometry. The three different types of tissue samples, flash frozen, OCT embedded tissue and LCM sections were processed using optimized methods for each sample type. Flash frozen tissues were extracted and directly processed using the trifluoroethanol method (5; 6). For the OCT embedded tissue, the mounting media was washed with PBS prior to protein extraction using the TFE protocol prior to liquid chromatography mass spectrometry (LC/MS/MS) as previously described (5; 6). In this approach, pancreas tissues were homogenized in lysis buffer comprising of 50% TFE in 50mM ammonium bicarbonate, pH 8.3, and incubated on ice for 30 minutes at 4°C to solubilize protein. The mixture was fully homogenized by probe sonication using 20 second cycles, 5 times on ice. Next, to maximize protein solubilization, the homogenized mixtures were heated at 60°C for 30 minutes, then sonicated again prior to centrifugation for 15 min at 10,000 x g to remove

insoluble material. The supernatant comprising of solubilized protein was collected and the concentration determined using the BCA assay.

Fifty micrograms of protein from each sample were heat-denatured at 95 °C for 5 minutes before adding 10 mM DTT and heating again at 95 °C for 5 minutes. The reduced samples were cooled to room temperature before alkylation using 15mM iodoacetamide for 30 minutes. The final concentration of TFE was reduced from 50% to 5% in a final volume of 500 µl using 50mM ammonium bicarbonate before digestion with trypsin at a 20:1 protein-to protease ratio at 37°C for 18 hrs. Trypsinization was stopped by the addition of formic acid before centrifugation at 20,000 x g for 10 minutes. The peptides were desalted by solid phase extraction using C18 columns and eluted with 80% acetonitrile in 0.1% formic acid. Eluted peptides were dried in a SpeedVac and stored at -80°C before further analysis. The tryptic peptides were solubilized in normalized volumes of 0.1% formic acid and their concentrations determined using a NanoDrop spectrophotometer. The peptide concentrations were adjusted to 0.5 µg/µl for all the samples before using 2 µg of each for analysis. Laser captured microdissected islet samples were processed for LC/MS/MS as previously described (7).

In complementary experiments, immunoprecipitation was performed on pancreas protein lysates using the anti-VP1 DAKO Clone 5D8/1 monoclonal antibody, using standard methods. Briefly, Protein A/G beads (Thermo Fisher) were added to concentration normalized samples and rotated overnight at 4°C. Samples were centrifuged at 3,000 rpm for 5 min at 4°C to pellet the beads and the supernatants were discarded. The beads were washed three times with cold 1 x RIPA buffer and suspended in SDS-PAGE loading buffer and heated at 95℃ for 10 min, then subjected to SDS-PAGE separation. The separated proteins were stained with colloidal

Coomassie to visualize protein bands. The protein bands were excised and processed for Gel-LC/MS/MS as previously described (8).

To improve virus peptide identification rates and to test possible concordance with VP-1 immunostaining on tissue sections, we included in the analysis pancreas sections that were also selected based on positive VP1 signals by IHC on FFPE tissues, presence of HLA Class I hyper-expression and detection of insulin staining in residual beta cells using fresh frozen OCT embedded tissues, as determined by related efforts within the nPOD-Virus Group. Thirty-micrometer (30 µM) tissue slices adjacent to those that were positive for VP1, insulin, and HLA Class I hyper-expression and corresponding negative controls were cut from OCT embedded tissues. Proteins were isolated from the sections and processed for LC/MS/MS using the TFE/ABC approach. For these experiments, we analyzed 18 new nPOD cases that included 8 ND donors, 3 AAb+ donors, and 7 donors with type 1 diabetes. The demographics and disease phenotypes for these cases are summarized in Supplementary Table 1 and 2 of Nyalwidhe et al, 2024; https://www.medrxiv.org/content/10.1101/2024.10.24.24315944v1.full .

Liquid Chromatography Mass Spectrometry Data Acquisition. Most LC-MS/MS analyses were performed on a Q-Exactive Orbitrap mass spectrometer (MS) (Thermo Fisher) and a Tribrid Orbitrap Fusion Lumos MS (Thermo Fisher), coupled on-line to a nanoflow LC system (Easy Nano 1200, Thermo Fisher). For these analyses we utilized data dependent acquisition (DDA) and data independent acquisition (DIA) methods [20-21]. A limited number of analyses were performed on a 5600 Triple TOF MS (Sciex), and on a Q-TRAP 4000 mass spectrometer (Sciex) coupled to an Eksigent nano-LC system (Sciex). For proteomic analysis, tryptic peptides were resolved using at a normalized concentration of 0.5 µg/µl 0.1% formic acid for each sample prior to

LC/MS analysis. Four microliters of the reconstituted peptides corresponding to 2 μg of the peptides were delivered to a trap column (Acclaim PepMap 100 C18, dimensions 0.1 × 2 cm) at a flow rate of 10 μl/min for 10 min using 0.1% formic acid. The trapped peptides were washed, equilibrated and transferred to a 50 cm, 75 μM inner diameter Thermo Scientific™ EASY-Spray C18 analytical column. Peptides were fractionated and injected into the MS using a 110-min gradient from 2% to 32% solvent B (0.1% FA, 80% in acetonitrile, ACN) at a flow rate of 300 nL/min. The acquisition parameters for the MS experiments are provided in the Supplementary Data.

Data Processing and Database Searching. Thermo RAW files were processed using the latest version of Xcalibur (Thermo Fisher Scientific). Mass spectral peaks were automatically identified by the software using default settings and filtered to include only peaks with charge states between +2and +7 m/z. Spectral data were converted into .mgf files using MSconvert (ProteoWizard) or Mascot Distiller (Matrix Science, London, UK). The data were searched for peptide identification using Mascot (Matrix Science, London UK). Tandem MS data were searched against the latest version of a combined Human (taxonomy ID 9606) and Enterovirus (taxonomy ID 12059) databases downloaded from the latest UniProt database (https://www.uniprot.org/).

The following search parameters were used: precursor mass tolerance was set to 10 ppm and fragment mass tolerance was set as 0.08 Da. Enzyme was set as trypsin with two missed cleavages permitted. Carbamidomethylation of cysteine was set as a fixed modification and oxidation of methionine, deamidation of asparagine and glutamine, and protein N-terminal acetylation (protein N-Term) were set as variable modifications. The Mascot decoy database function was enabled, and the false

discovery rate was set at < 1%, while individual ions scores >13 indicated identity or extensive homology (p<0.05). Only bold red peptides were considered in the protein identifications. A bold red match is the highest scoring match to a particular query listed under the highest scoring protein containing that match. Complementary targeted analyses were performed using Pinnacle (Optys Tech Corporation) and Scaffold DIA (Proteome Software).

Bioinformatics Analysis. BLAST annotation of identified viral proteins was performed using the BLASTP (https://blast.ncbi.nlm.nih.gov/). Sequence similarity search with an E-value threshold set at 1E-03 was carried out without taxonomical restriction against non-redundant protein sequences in the National Center for Biotechnology Information (NCBI) database. A search of the conserved domain (CD) of proteins with the Batch CD-Search tool of NCBI server was performed to support BLAST annotations.

Detection of enterovirus RNA in pancreas and lymphoid tissues of organ donors with type 1 diabetes

Jutta E Laiho[1*], Sami Oikarinen[1*], Sofia Morfopoulou[2], Maarit Oikarinen[1], Ashlie Renner[3], Daniel Depledge[4], Matthew C Ross[3,5], Ivan C Gerling[6], Judith Breuer[7], Joseph F Petrosino[3,5], Vincent Plagnol[8], Alberto Pugliese[9], Antonio Toniolo[10], Richard E Lloyd[3], Heikki Hyöty[1,11,12] for the JDRF nPOD-Virus Group

Research design and methods

Organ donors and tissues

We examined tissue samples from cadaveric organ donors collected by nPOD. As part of the coordinated efforts of the nPOD-Virus Group, we investigated tissues from 167 organ donors: 71 donors with type 1 diabetes, of which 35 had residual insulin containing islets (T1D-ICI) and 36 only had insulin-deficient islets (T1D-IDI); 22 islet autoantibody (AAb) positive donors without diabetes considered at increased risk for type 1 diabetes, of whom 15 donors expressed a single autoantibody (AAb+), and 7 donors had ≥2 autoantibodies (AAb++). Finally, 74 autoantibody-negative donors without diabetes were included as a control group (ND). Demographic information for each group is summarized in **Table 1** (Laiho et al, accompanying paper). Detailed donor information is provided in **ESM Table 1** (Laiho et al, accompanying paper). The standardised collection protocol for the tissues analysed is described in Campbell-Thompson et al (9).

Briefly, alternating pancreas slices were used for fixed paraffin and frozen blocks. From select cases, other organs were recovered: spleen, pancreatic and non-pancreatic lymph nodes, live cryopreserved lymphoid cells, duodenal mucosa. The frozen samples were stored in liquid nitrogen. All samples were de-identified and obtained by nPOD through its partnership organ procurement organizations, after consent for organ donation and research was obtained from family members. Frozen samples were shipped by air to participating laboratories using small liquid nitrogen containers. On arrival, samples were stored at -70°C until used.

Five different laboratories performed independent assays using diverse methodologies to detect traces of enteroviruses or other microbes in pancreas and other tissues. A goal of the nPOD-Virus group was to approach the question about viral aetiology of type 1 diabetes and explore what type of viruses may be present, and if so, potentially associated with disease. To this end, we implemented two

unbiased discovery approaches for microbes, based on two different RNA-Seq methods. In addition, based on pre-existing evidence of an association of type 1 diabetes with enterovirus infections, we employed enterovirus specific RT-PCR assays, and enterovirus propagation in cell cultures of the virus, followed by RT-PCR and as well as enterovirus capsid protein staining. Samples from the donors were distributed to the five participating laboratories according to the protocol shown in **Fig. 1** (Laiho et al, 2024, an accompanying manuscript)

Unbiased discovery of microbes

RNA-Seq studies were performed on pancreas samples in two laboratories at the University College London (UCL), London, UK, and at the Baylor College of Medicine (BCM), Houston, USA. Based on sample availability for coordinated studies, RNA-Seq analyses were performed on pancreas samples from 63 nPOD donors: 6 T1D-ICI, 10 T1D-IDI, 4 AAb++, 12 AAb+ and 21 ND donors. Of the above 63 donors, 29 were analysed in both laboratories (11 T1D-ICI, 1 T1D-IDI, 4 AAb++, 4 AAb+, 9 ND).

RNA-Seq analyses at UCL

Over four years, UCL sequenced frozen pancreas samples from 33 nPOD cases (12 T1D-ICI, 1 T1D-IDI, 4 AAb+, 4 AAb++ and 12 ND). We developed the methodology in four steps (described below) and used several extraction and library preparation approaches, to maximize sensitivity. Initial negative results motivated the development of a specific sequence capture method (10) to enrich for enteroviral sequences and adding the analysis of laser captured islet RNA to further increase sensitivity (1). The RNA obtained was then subjected to Illumina high throughput RNA sequencing.

In step I (first pilot stage), we examined pancreas from 3 T1D-ICI, 1 T1D-IDI and 2 ND cases, based on availability of optimal samples for RNA-Seq. Disease duration ranged from 4 to 28 years. *Step II* investigated tissues from donors with shorter disease duration to minimize the time between sample collection and T1D onset [4 T1D-ICI cases (disease duration range: 1-5 years), 4 AAb++ and 3 AAb+ cases]. In *step III* we examined pancreas from 4 T1D-ICI cases with enterovirus VP1 immuno-positivity by immunohistochemistry and HLA class I hyperexpression, along with 5 ND donors (for details, see immunohistochemistry results in the accompanying publication Rodriguez-Calvo et al). From one donor with type 1 diabetes, two samples were analysed. Finally, in *step IV* we examined laser micro-dissected islets from 6 T1D-ICI, 4 autoantibody-positive (3 AAb++, 1 AAb+) and 6 ND donors. From one donor with type 1 diabetes, two samples were analysed.

In *steps I and II*, total RNA was isolated using Illumina GAIIx (step I) or the Illumina HiSeq2500 (step II), followed by a poly(A) selection step for mRNA. In *steps III and IV* we used the Agilent SureSelect system to enrich the potential enteroviral sequences in pancreatic samples. RNA extraction was performed as described (4). For double-stranded (ds) cDNA generation, we used a protocol optimized for RNA viruses (10; 11). The ds-cDNA was sheared, and libraries prepared as per the SureSelect protocol v1.4. Enrichment for enteroviral sequences was performed using a set of 120-mer biotinylated RNA oligonucleotides prior to indexing and sequencing on different Illumina platforms (MiSeq, HiSeq, NextSeq). The bait set (RNA oligonucleotides) was designed using an in-house script written for an EU-funded project aimed at using SureSelect in a pathogen diagnostic setting (PathSeek). The bait set hybridized against all members of the *Enterovirus A* species (n=363 probes), *B* species (n=176) and *C* species (n=303), based on sequences were available in Genbank at the time of

design (15 May 2013). Up to 8 mismatches in a 120-mer oligo was accepted to still enable capture of the targeted sequence, ensuring enterovirus detection provided these shared a reasonable degree of similarity.

Positive control experiment for sequence capture

As positive control, ULC sequenced pancreatic tissue samples that were spiked in at different dilutions (10^-4 to 10^-8 range) of coxsackie B virus 1 (CVB1) and a negative control, to assess the sensitivity of the sequence capture method prior to its use (**ESM data 1)** (Laiho et al, accompanying paper).

<u>*Metagenomic whole genome shotgun sequencing at BCM.*</u>

BCM performed metagenomic whole genome shotgun (WGS) sequencing from 60 nPOD frozen pancreas samples (16 T1D-ICI, 10 T1D-IDI, 12 AAb+, 4 AAb++ and 18 ND). Total pancreatic nucleic acids were extracted using the MagMax Viral RNA Isolation Kit (Cat # AM1939, Thermo Fisher, Waltham, MA), without DNAse to prevent DNA removal. Extracted viral RNA was reverse transcribed using SuperScript II RT (Cat # 18064014, Thermo Fisher) and random hexamers. After short molecule and random hexamer removal with ChargeSwitch (Cat # CS12000, Thermo Fisher), molecules were amplified and tagged with a 12 base-pair barcode tag containing a V8A2 semi-random primer (BC12-V8A2 construct using AccuPrimeTM Taq polymerase and cleaned with ChargeSwitch kit). Tags were attached via a barcoded, semi-random primer construct resulting in dual barcoded (same barcode on both sides) amplified fragments. The indexes used were 12 bp Golay Barcodes. Separate negative controls were introduced during extraction, amplification, and library preparation steps. We performed a single WGS library prep per sequencing lane

(without shearing) of pooled, pre-barcoded samples to minimize carry-over, as each lane only had a single index. Since all samples carried secondary internal barcodes, they were not subject to carry-over or cross-bleed that sometimes is observed from run to run with library indexes using the Illumina platform. The size of the library was verified via bioanalyzer to ensure appropriate range for the platform (~200-1000 bp). The library was then loaded in an Illumina HiSeq2000 (Illumina, Carlsbad, CA) and sequenced using the 2x100bp chemistry at the Human Genome Sequencing Center, BCM. Reads were demultiplexed into a sample bin using the barcode prefixing read-1 and read- 2, allowing zero mismatches. Demultiplexed reads were further processed by trimming off barcodes, semi-random primer sequences, and Illumina adapters. This process utilized a custom demultiplexer and the BBDuk algorithm included in BBMap53.

Bioinformatics analysis and community profiling at UCL and BCM

A dual analytic approach was implemented. Data was first analysed in unbiased manner, assuming no prior knowledge of potential pathogens and characterizing the full species profile for each sample. In addition, data was specifically searched for enteroviral sequences. PCR duplicates were removed with an in-house script that collapses read pairs by sequence identity using 90% of the sequence as signature. We removed low quality and low complexity sequences with PrinSeq (12) and human sequences with Novoalign (version V2.07.13 - human reference genome GRCh37) followed by BLASTn (13). High quality contigs of at least 200bp length were *de novo* assembled with Velvet (14). Contigs and the unassembled reads were annotated with BLASTx (default parameters) and a custom protein database consisting of viral, human microbiome bacterial, human and mouse RefSeq proteins (October 2013

version). Coxsackievirus proteins that were not present in the RefSeq collection were added to the database. To search specifically for enterovirus sequences, we aligned (Novoalign V2.07.13) quality-controlled reads simultaneously to the genomes of enteroviruses from species A, B and D (NC_001612, NC_001472, NC_001430). This search was repeated using all enterovirus full genomes from GenBank (221 genomes, January 2020) and Bowtie2 (15). We employed metaMix 0.1 (16), which is used in clinical diagnostics for pathogen detection in brain biopsies from patients with encephalitis of unknown cause (17-22), to characterize the species that are present in each sample. The read support parameter cutoff for a species to be retained in the profile, was ten reads.

Targeted enterovirus detection by RT-PCR

The presence of enterovirus RNA was assessed in tissues from 141 nPOD organ donors using a sensitive RT-PCR assay. Frozen pancreas samples from 137 nPOD organ donors (32 T1D-ICI, 34 T1D-IDI, 7 AAb++, 15 AAb+, 49 ND) were analysed in two laboratories at Tampere University, Finland, and in the Department of Molecular Virology and Microbiology, BCM, Houston, TX, USA. Based on sample availability, the Tampere laboratory also examined frozen spleen samples from 97 organ donors (19 T1D-ICI, 23 T1D-IDI, 7 AAb++, 12 AAb+ and 36 ND), pancreatic lymph node (PLN) samples from 8 organ donors (3 T1D-ICI, 2 T1D-IDI and 3 AAb++), and duodenum samples from 65 organ donors (9 T1D-ICI, 22 T1D-IDI, 5 AAb++, 8 AAb+ and 21 ND).

In Tampere, RNA was extracted from frozen tissue using the Viral RNA Kit (Qiagen, Hilden, Germany) and samples were analysed with a quantitative real-time RT-PCR method (23). In the BCM laboratory, pancreatic RNA was extracted with the MagMax

Viral RNA Isolation Kit (Invitrogen; Thermo Fischer). RNA was converted to cDNA with Superscript III RT (Invitrogen) according to the manufacturer's directions, with random primers. PCR was carried out with SYBR-Green PCR master mix (Invitrogen) using the same primers as in Tampere (24). PCR included a denaturation step (95 °C for 10 min) followed by 50 cycles of 95 °C for 30 s and 60 °C for 60 s. In both laboratories, positive RT-PCR signals were confirmed by sequencing the PCR amplicon and samples were considered positive only if an enterovirus sequence was obtained.

The degree of RNA degradation was analysed in selected pancreas, spleen and duodenum samples, using Agilent Fragment Analyzer.

Enterovirus propagation in cell culture prior to RNA detection by RT-PCR

Enterovirus propagation in cell cultures was carried out for spleen samples at the University of Insubria, Varese, Italy, to amplify the virus prior to RT-PCR assays and immunostaining. For this approach we selected spleen samples since they do not contain enzymes that can affect cultured cells. Donors were selected according to the availability of live spleen cell suspensions for both controls and T1D donors. Snap frozen spleen tissue was also tested in the form of spleen homogenates. We could examine samples from 69 donors (16 T1D-ICI, 9 T1D-IDI, 2 AAb++, 6 AAb+, 36 ND). A published procedure for detecting persistent enterovirus infections (24; 25) was followed with minor modifications. Briefly, to enrich for virus nPOD spleen samples (live cells or tissue homogenates) were co-cultured in T-25 flasks with five different human cell lines AV3, RD, 1.1B4, VC3, HEK-293 (European Collection of Authenticated Cell Cultures, Porton Down, UK) that express a wide range of enterovirus receptors. Human cell lines were grown in DME/F12 medium

supplemented with penicillin/gentamicin and with 10% heat inactivated FBS (Gibco; Thermo Fisher, Rodano, Italy). Cultured cells were checked monthly for mycoplasma contamination (MycoAlert Plus Mycoplasma kit; Euroclone-Lonza, Pero, Italy). For immunofluorescent detection of enterovirus VP1 antigen, cell cultures were prepared in Millicell EZ 4-well glass slides (Merck, Vimodrone, Italy) as described below. At the third passage, the supernatant of cell cultures was used for RNA extraction and RT-PCR. Extracted RNA was reverse transcribed and enterovirus-specific end-point PCR assays were performed using five different primer sets. Capillary electrophoresis (Agilent 2100 Bioanalyzer, Milano, Italy) was used to detect the precise size of amplicons whose sequences were obtained by the Sanger method. For indirect immunofluorescence, cell monolayers were fixed in PBS containing 4% paraformaldehyde. Enterovirus-infected cells were spotted by staining with two different mouse monoclonal antibodies against the VP1 enteroviral capsid antigen (9D5 from Merck; 6-E9/2 from Creative Diagnostics). The two antibodies bind to distinct stretches of the VP1 protein. Both recognize acute and persistent enterovirus infection in cultured cells, are devoid of neutralizing activity, and are specific for a vast spectrum of EV types. The 9D5 antibody binds to the consensus motif SIGNAYSMFYDG (26) while 6-E9/2 recognizes the same epitope of the 5D8/1 antibody (2). Alexa Fluor 488-goat anti-mouse IgG was used as secondary antibody.

Statistical analysis

Statistical analyses were performed using SPSS 25.0 for Windows. Frequency comparisons was performed with the Pearson's X^2 and Fisher's exact tests. When comparing donor groups, the significant p values were corrected for multiple

comparisons by multiplying the raw p-value by the number of comparisons made (Bonferroni's correction).

References

1. Richardson SJ, Leete P, Bone AJ, Foulis AK, Morgan NG: Expression of the enteroviral capsid protein VP1 in the islet cells of patients with type 1 diabetes is associated with induction of protein kinase R and downregulation of Mcl-1. Diabetologia 2013;56:185-193
2. Richardson SJ, Leete P, Dhayal S, Russell MA, Oikarinen M, Laiho JE, Svedin E, Lind K, Rosenling T, Chapman N, Bone AJ, n PODVC, Foulis AK, Frisk G, Flodstrom-Tullberg M, Hober D, Hyoty H, Morgan NG: Evaluation of the fidelity of immunolabelling obtained with clone 5D8/1, a monoclonal antibody directed against the enteroviral capsid protein, VP1, in human pancreas. Diabetologia 2014;57:392-401
3. Richardson SJ, Willcox A, Bone AJ, Foulis AK, Morgan NG: The prevalence of enteroviral capsid protein vp1 immunostaining in pancreatic islets in human type 1 diabetes. Diabetologia 2009;52:1143-1151
4. Richardson SJ, Rodriguez-Calvo T, Gerling IC, Mathews CE, Kaddis JS, Russell MA, Zeissler M, Leete P, Krogvold L, Dahl-Jorgensen K, von Herrath M, Pugliese A, Atkinson MA, Morgan NG: Islet cell hyperexpression of HLA class I antigens: a defining feature in type 1 diabetes. Diabetologia 2016;59:2448-2458
5. Burch TC, Morris MA, Campbell-Thompson M, Pugliese A, Nadler JL, Nyalwidhe JO: Proteomic Analysis of Disease Stratified Human Pancreas Tissue Indicates Unique Signature of Type 1 Diabetes. PLoS One 2015;10:e0135663
6. Wang H, Qian WJ, Mottaz HM, Clauss TR, Anderson DJ, Moore RJ, Camp DG, 2nd, Khan AH, Sforza DM, Pallavicini M, Smith DJ, Smith RD: Development and evaluation of a micro- and nanoscale proteomic sample preparation method. J Proteome Res 2005;4:2397-2403
7. Nyalwidhe JO, Grzesik WJ, Burch TC, Semeraro ML, Waseem T, Gerling IC, Mirmira RG, Morris MA, Nadler JL: Comparative quantitative proteomic analysis of disease stratified laser captured microdissected human islets identifies proteins and pathways potentially related to type 1 diabetes. PLoS One 2017;12:e0183908
8. Guo X, Ward MD, Tiedebohl JB, Oden YM, Nyalwidhe JO, Semmes OJ: Interdependent phosphorylation within the kinase domain T-loop Regulates CHK2 activity. J Biol Chem 2010;285:33348-33357
9. Campbell-Thompson ML, Montgomery EL, Foss RM, Kolheffer KM, Phipps G, Schneider L, Atkinson MA: Collection protocol for human pancreas. J Vis Exp 2012:e4039
10. Depledge DP, Palser AL, Watson SJ, Lai IY, Gray ER, Grant P, Kanda RK, Leproust E, Kellam P, Breuer J: Specific capture and whole-genome sequencing of viruses from clinical samples. PLoS One 2011;6:e27805

11. Brown JR, Roy S, Ruis C, Yara Romero E, Shah D, Williams R, Breuer J: Norovirus Whole-Genome Sequencing by SureSelect Target Enrichment: a Robust and Sensitive Method. J Clin Microbiol 2016;54:2530-2537
12. Schmieder R, Edwards R: Quality control and preprocessing of metagenomic datasets. Bioinformatics 2011;27:863-864
13. Altschul SF, Gish W, Miller W, Myers EW, Lipman DJ: Basic local alignment search tool. J Mol Biol 1990;215:403-410
14. Zerbino DR, Birney E: Velvet: algorithms for de novo short read assembly using de Bruijn graphs. Genome Res 2008;18:821-829
15. Langmead B, Salzberg SL: Fast gapped-read alignment with Bowtie 2. Nat Methods 2012;9:357-359
16. Morfopoulou S, Plagnol V: Bayesian mixture analysis for metagenomic community profiling. Bioinformatics 2015;31:2930-2938
17. Brown JR, Morfopoulou S, Hubb J, Emmett WA, Ip W, Shah D, Brooks T, Paine SM, Anderson G, Virasami A, Tong CY, Clark DA, Plagnol V, Jacques TS, Qasim W, Hubank M, Breuer J: Astrovirus VA1/HMO-C: an increasingly recognized neurotropic pathogen in immunocompromised patients. Clin Infect Dis 2015;60:881-888
18. de Vries JJC, Brown JR, Fischer N, Sidorov IA, Morfopoulou S, Huang J, Munnink BBO, Sayiner A, Bulgurcu A, Rodriguez C, Gricourt G, Keyaerts E, Beller L, Bachofen C, Kubacki J, Samuel C, Florian L, Dennis S, Beer M, Hoeper D, Huber M, Kufner V, Zaheri M, Lebrand A, Papa A, van Boheemen S, Kroes ACM, Breuer J, Lopez-Labrador FX, Claas ECJ: Benchmark of thirteen bioinformatic pipelines for metagenomic virus diagnostics using datasets from clinical samples. J Clin Virol 2021;141:104908
19. Duncan CJ, Mohamad SM, Young DF, Skelton AJ, Leahy TR, Munday DC, Butler KM, Morfopoulou S, Brown JR, Hubank M, Connell J, Gavin PJ, McMahon C, Dempsey E, Lynch NE, Jacques TS, Valappil M, Cant AJ, Breuer J, Engelhardt KR, Randall RE, Hambleton S: Human IFNAR2 deficiency: Lessons for antiviral immunity. Sci Transl Med 2015;7:307ra154
20. Lum SH, Turner A, Guiver M, Bonney D, Martland T, Davies E, Newbould M, Brown J, Morfopoulou S, Breuer J, Wynn R: An emerging opportunistic infection: fatal astrovirus (VA1/HMO-C) encephalitis in a pediatric stem cell transplant recipient. Transpl Infect Dis 2016;18:960-964
21. Morfopoulou S, Brown JR, Davies EG, Anderson G, Virasami A, Qasim W, Chong WK, Hubank M, Plagnol V, Desforges M, Jacques TS, Talbot PJ, Breuer J: Human Coronavirus OC43 Associated with Fatal Encephalitis. N Engl J Med 2016;375:497-498
22. Morfopoulou S, Mee ET, Connaughton SM, Brown JR, Gilmour K, Chong WK, Duprex WP, Ferguson D, Hubank M, Hutchinson C, Kaliakatsos M, McQuaid S, Paine S, Plagnol V, Ruis C, Virasami A, Zhan H, Jacques TS, Schepelmann S, Qasim W, Breuer J: Deep sequencing reveals persistence of cell-associated mumps vaccine virus in chronic encephalitis. Acta Neuropathol 2017;133:139-147
23. Honkanen H, Oikarinen S, Pakkanen O, Ruokoranta T, Pulkki MM, Laitinen OH, Tauriainen S, Korpela S, Lappalainen M, Vuorinen T, Haapala AM, Veijola R, Simell O, Ilonen J, Knip M, Hyoty H: Human enterovirus 71 strains in the background population and in hospital patients in Finland. J Clin Virol 2013;56:348-353
24. Genoni A, Canducci F, Rossi A, Broccolo F, Chumakov K, Bono G, Salerno-Uriarte J, Salvatoni A, Pugliese A, Toniolo A: Revealing enterovirus infection in chronic human disorders: An integrated diagnostic approach. Sci Rep 2017;7:5013

25. Krogvold L, Genoni A, Puggioni A, Campani D, Richardson SJ, Flaxman CS, Edwin B, Buanes T, Dahl-Jorgensen K, Toniolo A: Live enteroviruses, but not other viruses, detected in human pancreas at the onset of type 1 diabetes in the DiViD study. Diabetologia 2022;65:2108-2120
26. Maccari G, Genoni A, Sansonno S, Toniolo A: Properties of Two Enterovirus Antibodies that are Utilized in Diabetes Research. Sci Rep 2016;6:24757

Funding:
Breakthrough T1D (formally JDRF) JDRF-25-2012-516
Participants:
Alberto Pugliese, M.D., Arthur Riggs Diabetes & Metabolism Research Institute, City of Hope, Duarte, CA, USA Principal Investigator
Mark Atkinson, Ph.D., University of Florida Sr. Advisor
Martha Campbell-Thompson, Ph.D. University of Florida Investigator
Nora Chapman, Ph.D., University of Nebraska Investigator
Ken Coppieters, Ph.D., University of Ghent Investigator
Francesco Dotta, M.D., University of Siena Investigator
George Eisenbarth, M.D., Ph.D., Barbara Davis Center for Childhood Diabetes Sr. Advisor
Ricardo Ferreira, Ricardo, Ph.D., JDRF/WT Diabetes & Inflammation Laboratory Investigator (Post-Doc)
Gun Frisk, Ph.D., University of Uppsala Investigator
Roberto Gianani, M.D., Barbara Davis Center for Childhood Diabetes Investigator
Ivan Gerling, Ph.D, University of Tennessee Investigator
Dirk Homann, M.D., University of Colorado at Denver Investigator
Heikki Hyoty, Ph.D., Tampere University, Finland Investigator
Richard Lloyd, Ph.D. Baylor College of Medicine Investigator
John Kaddis, Ph.D. City of Hope National Medical Center Investigator
Sally Kent, Ph.D. University of Massachusetts Investigator
Noel Morgan, Ph.D., University of Exeter, UK Investigator
Jerry Nadler, M.D., Ph.D., East Virginia Medical School Investigator
Margaret Morris Fear, Ph.D., East Virginia Medical School Investigator
Julius Nyalwhide, Ph.D. East Virginia Medical School Investigator
Maarit Oikarinen, Ph.D., Tampere University, Finland Research Scientist
Vincent Plagnol, Ph.D. University College of London Investigator
Joseph Petrosino, Ph.D. Baylor College of Medicine Investigator
Sarah Richardson, Ph.D. University of Exeter, UK Investigator (Post-Doc)
Suparna Sarkar, Barbara Davis Center for Childhood Diabetes Investigator
Daris Schneider, La Jolla Inst. Allergy & Immunology Investigator (Post-Doc)
Larissa Thackray, Ph.D. Washington University Investigator
Antonio Toniolo, M.D. University of Insubria *Investigator*
Herbert Virgin, Washington University Investigator
Matthias Von Herrath, La Jolla Inst. Allergy & Immunology Investigator

Breakthrough T1D (formally JDRF) JDRF-3-SRA-2017-492-A-N
Participants:
Alberto Pugliese, Arthur Riggs Diabetes & Metabolism Research Institute, City of Hope, Duarte, CA, USA Principal Investigator
Richard Lloyd, Baylor College of Medicine, USA.
Margaret Morris, Eastern Virginia Medical School, USA
Roberto Mallone, INSERM, Paris, France
Malin Flodström Tullberg, Karolinska Institutet, Sweden

Matthias von Herrath, La Jolla Institute for Allergy and Immunology, USA
Jerry Nadler, Eastern Virginia Medical School, USA
Julius Nyalwidhe, Eastern Virginia Medical School, USA
Maria Teresa Rodriguez Calvo, La Jolla Institute for Allergy and Immunology.
Sally Kent, University of Massachusetts Medical School, USA
Antonio Toniolo, University of Insubria, Italy
Kathrin Maedler, University of Bremen, Germany
Marc Horwitz, University of British Columbia, Canada
Noel Morgan, University of Exeter Medical School, UK
Sarah Richardson, University of Exeter Medical School, UK
Mark Atkinson, University of Florida, USA
Ivan Gerling, The University of Tennessee Health Science Center, USA
Heikki Hyoty, *Tampere University, Finland*
Isaac Snowhite, University of Miami School of Medicine, USA
Filippo Canducci, University of Insubria, Italy
Alessandro Salvatoni, University of Insubria*, Italy*

***Additional members* (some participated in discussions, but their participation in the group is more informal).**
Soile Tuomela PhD (Karolinska Institutet, Sweden)
Ben Giepmans PhD (University of Groningen, Netherlands)
Varpu Marjomaki PhD (University of Jyväskylä, Finland)
